# Vaccine-induced protection against SARS-CoV-2 requires IFN-γ-driven cellular immune response

**DOI:** 10.1038/s41467-023-39096-y

**Published:** 2023-06-10

**Authors:** Xiaolei Wang, Terrence Tsz-Tai Yuen, Ying Dou, Jingchu Hu, Renhao Li, Zheng Zeng, Xuansheng Lin, Huarui Gong, Celia Hoi-Ching Chan, Chaemin Yoon, Huiping Shuai, Deborah Tip-Yin Ho, Ivan Fan-Ngai Hung, Bao-Zhong Zhang, Hin Chu, Jian-Dong Huang

**Affiliations:** 1 School of Biomedical Sciences, Li Ka Shing Faculty of Medicine, The University of Hong Kong, Pokfulam, Hong Kong Special Administrative Region People’s Republic of China; 2 Clinical Oncology Center, The University of Hong Kong-Shenzhen Hospital, Shenzhen, Guangdong Province China; 3 Department of Microbiology, School of Clinical Medicine, Li Ka Shing Faculty of Medicine, The University of Hong Kong, Pokfulam, Hong Kong Special Administrative Region People’s Republic of China; 4 Department of Medicine, School of Clinical Medicine, Li Ka Shing Faculty of Medicine, The University of Hong Kong, Pokfulam, Hong Kong Special Administrative Region People’s Republic of China; 5 Department of Infectious Disease and Microbiology, The University of Hong Kong-Shenzhen Hospital, Shenzhen, Guangdong Province People’s Republic of China; 6 CAS Key Laboratory of Quantitative Engineering Biology, Shenzhen Institute of Synthetic Biology, Shenzhen Institute of Advanced Technology, Chinese Academy of Sciences, Shenzhen, 518055 China; 7 State Key Laboratory of Emerging Infectious Diseases, The University of Hong Kong, Pokfulam, Hong Kong Special Administrative Region People’s Republic of China; 8 Centre for Virology, Vaccinology and Therapeutics, Hong Kong Science and Technology Park, Hong Kong, Hong Kong Special Administrative Region People’s Republic of China; 9 Guangdong-Hong Kong Joint Laboratory for RNA Medicine, Sun Yat-Sen University, Guangzhou, China; 10 Clinical Oncology Center, Shenzhen Key Laboratory for cancer metastasis and personalized therapy, The University of Hong Kong-Shenzhen Hospital, Shenzhen, China

**Keywords:** Vaccines, Cellular immunity, SARS-CoV-2, B cells

## Abstract

The overall success of worldwide mass vaccination in limiting the negative effect of the COVID-19 pandemics is inevitable, however, recent SARS-CoV-2 variants of concern, especially Omicron and its sub-lineages, efficiently evade humoral immunity mounted upon vaccination or previous infection. Thus, it is an important question whether these variants, or vaccines against them, induce anti-viral cellular immunity. Here we show that the mRNA vaccine BNT162b2 induces robust protective immunity in K18-hACE2 transgenic B-cell deficient (μMT) mice. We further demonstrate that the protection is attributed to cellular immunity depending on robust IFN-γ production. Viral challenge with SARS-CoV-2 Omicron BA.1 and BA.5.2 sub-variants induce boosted cellular responses in vaccinated μMT mice, which highlights the significance of cellular immunity against the ever-emerging SARS-CoV-2 variants evading antibody-mediated immunity. Our work, by providing evidence that BNT162b2 can induce significant protective immunity in mice that are unable to produce antibodies, thus highlights the importance of cellular immunity in the protection against SARS-CoV-2.

## Abstract (web summary)

Protective immunity against SARS-CoV-2 relies on both antibodies and a T cell dependent response, however, direct experimental evidence for the contribution of cellular immunity is limited. Here authors present a mouse model that is susceptible to SARS-CoV-2 and lacks B cells to demonstrate the emergence of efficient cellular immune response against SARS-CoV-2 upon vaccination or viral challenge.

## Introduction

The Coronavirus Disease 2019 (COVID-19) pandemic continues as of August 2022. Vaccines are believed to be the most effective way to build herd immunity among populations across the globe. COVID-19 vaccines such as BNT162b2 have been shown to greatly mitigate the mortality of severe acute respiratory syndrome coronavirus 2 (SARS-CoV-2) infection^1^. These vaccines can induce robust immunity against SARS-CoV-2 in immunocompetent individuals^2,3^. In immunocompromised populations with a lack of, or insufficient production of antibodies, multiple human clinical data demonstrated that strong Spike-specific T cell responses were observed in BNT162b2 vaccinated patients who received anti-CD20 therapy^4–6^. However, the scientific evidence of whether vaccines can elicit sufficient protective immunity upon SARS-CoV-2 infection in this population is limited. Furthermore, the continuous emergence of SARS-CoV-2 variants such as Omicron and its sub-lineages has raised increasing concerns about the reduced efficacy of COVID-19 vaccines. Recent studies have demonstrated that Omicron and Omicron sub-lineages efficiently evade antibody-mediated immunity from vaccination or previous infection due to accumulating mutations in their spike proteins^7–10^. Nevertheless, the contribution of cellular immunity against infection of emerging variants of SARS-CoV-2 remains poorly defined in the context of vaccine-induced immunity.

B cell-deficient B6.129S2-Ighmtm1Cgn/J (μMT) mouse, which lacks the humoral arm of vaccine-induced immunity, is not only suitable for studying the contribution of cellular immunity elicited by BNT162b2 against SARS-CoV-2 variants that evade antibody-mediated immunity, but also an ideal mouse model to evaluate the protective efficacy of COVID-19 vaccines in individuals with humoral immunodeficiencies.

In this study, we systemically evaluate the efficacy of BNT162b2 vaccination in two murine μMT experimental models. The first model uses μMT mice in C57BL/6 J background that can be infected by Alpha (B.1.1.7), Omicron BA.1 (B.1.1.529.1) and BA.5.2 (B.1.1.529.5.2)^11^. The second model involves humanized μMT mice expressing transgenic human angiotensin-converting enzyme 2 (hACE2) receptor that increases the susceptibility of mice to SARS-CoV-2 in order to model moderate to severe COVID-19^12^.

We report here that BNT162b2 vaccination confers protective immunity against both Alpha, Omicron BA.1 and BA.5.2 infection in both μMT mouse models. The protection is associated with lower viral replication, reduced pulmonary pathology, and elevated IFN-γ production in blood, nasal turbinate and lung tissue of vaccinated μMT mice. IFN-γ depleting antibody significantly increases Omicron BA.1 replication in vaccinated μMT mice but not that of vaccinated WT mice, suggesting the IFN-γ signaling is a core component of the protective immunity in the vaccinated μMT mice. We further demonstrate that both CD4^+^ and CD8^+^ T cells contributed to the vaccine-induced protective immunity against SARS-CoV-2 in nasal turbinate and lung tissue from μMT mice. Together, our data suggest that T cell-mediated immunity provides significant immunity in the absence of antibody-mediated immunity in vaccinated μMT mice upon SARS-CoV-2 infection and retains the ability to respond to emerging variants of concern of SARS-CoV-2.

## Results

### BNT162b2 induces robust cellular responses in B lymphocyte-deficient mice

**Fig. 1:**
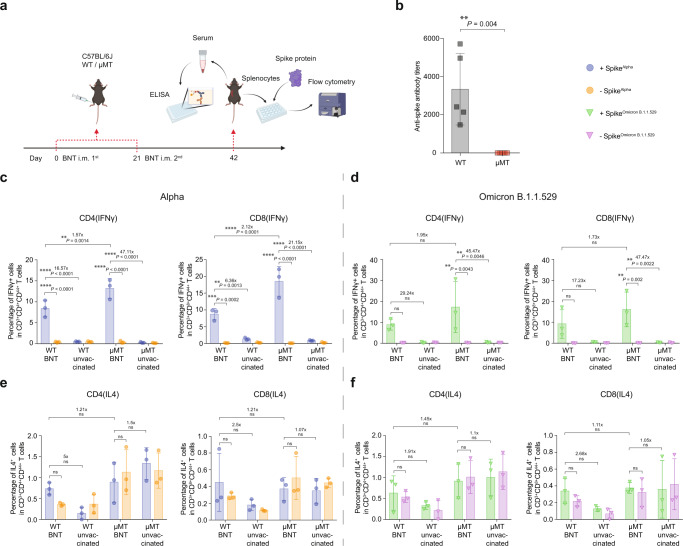
BNT162b2 induces robust cellular responses in mice C57BL/6 J μMT mice. **a** Schematic diagram of the vaccination scheme, ELISA, and flow cytometry. **b** Antibodies titer specific for spike protein of SARS-CoV-2 was measured by ELISA (*n* = 5). **c**–**f** The splenocytes were stimulated by spike proteins of SARS-CoV-2 Alpha or Omicron BA.1 variant and subjected to flow cytometry analysis for IFN-γ and IL-4 responses (*n* = 3). Data are presented as mean ± SD. Statistical significance was calculated using one-way ANOVA test (*p < 0.05, **p < 0.01, ***p < 0.001, ****p < 0.0001, ns = not significant). Figure 1a was created with BioRender.com.

To evaluate the cellular responses in μMT mice, we collected spleen tissues from vaccinated and unvaccinated C57BL/6 J wild type (WT) and μMT mice. The splenocytes were then stimulated with spike protein of SARS-CoV-2 (Fig. 1a). We first quantified antibodies titer against SARS-CoV-2 spike with ELISA and confirmed that antibody response was absent in BNT162b2-vaccinated μMT mice (Fig. 1b). Our flow cytometry analysis demonstrated that 13.2% of CD4^+^ and 18.5% of CD8^+^ T memory cells in spleen tissue of μMT mice immunized with BNT162b2 secreted IFN-γ upon stimulating with spike proteins of Alpha variant (Fig. 1c). In parallel, spike protein of Omicron BA.1 induced IFN-γ production in 17.3% of CD4^+^ and 16.1% of CD8^+^ T memory cells, suggesting robust induction of cellular responses upon stimulation of spike proteins from different SARS-CoV-2 variants (Fig. 1d). Importantly, the mean percentage of CD4^+^ and CD8^+^ T memory cells producing IFN-γ is higher in vaccinated μMT mice than in WT mice (Fig. 1c, d), suggesting a higher level of activation of cellular responses in the vaccinated μMT mice when compared to WT mice. In contrast, IL-4 expression, which reflects the differentiation of B cells into plasma cells^13^, was not stimulated by the spike proteins in either WT or μMT mice (Fig. 1e, f). Besides, it is reported that CD8^+^ T cells in B cell-deficient mice were skewed more toward effector phenotype during viral infection^14,15^. Interestingly, our data revealed that stimulation of spike protein of SARS-CoV-2 could also induce the skewed differentiation of CD8^+^ T cells into highly cytotoxic terminal effector cells in the spleen of vaccinated µMT mice (Fig. S1a). The proportion of long-lived CD8^+^ T memory cell precursors is, however, dramatically reduced in vaccinated µMT mice compared to that in vaccinated WT mice (Fig. S1a). Taken together, our results demonstrated that robust cellular immunity was elicited in μMT mice immunized with BNT162b2 in the absence of humoral immunity.

### BNT162b2 elicits significant protective immunity against SARS-CoV-2 Alpha and Omicron BA.1 in B lymphocyte-deficient mice

**Fig. 2:**
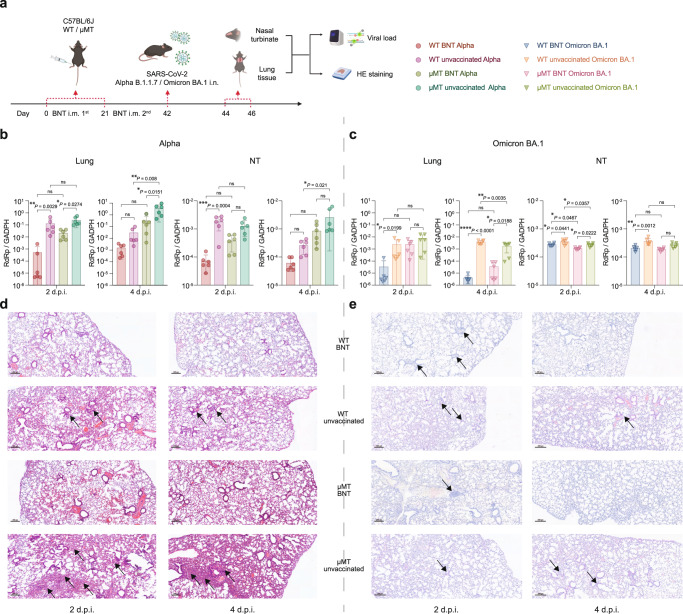
BNT162b2 elicits significant protective immunity in C57BL/6 J μMT mice. **a** Schematic diagram of vaccination, viral challenge, and pathological studies in C57BL/6 J model. **b**, **c** The viral loads in the lung and nasal turbinate (NT) of BNT162b2 vaccinated and unvaccinated C57BL/6 J WT/μMT mice at 2 d.p.i. and 4 d.p.i. (*n* = 6). **d**, **e** Representative images of the H&E-stained lung tissues of BNT162b2 vaccinated and unvaccinated C57BL/6 J WT/μMT mice challenged with Alpha (**d**) or Omicron BA.1 variant (**e**). Scale bar = 200 μm. Data are presented as mean ± SD. Statistical significance was calculated using one-way ANOVA test (*p < 0.05, **p < 0.01, ***p < 0.001, ****p < 0.0001, ns = not significant). Figure 2a was created with BioRender.com.

To assess the requirement of antibody-mediated immunity in the protection against SARS-CoV-2, we vaccinated C57BL/6 J WT and μMT mice with the BNT162b2 mRNA vaccine and infected the vaccinated mice with SARS-CoV-2 Alpha or Omicron BA.1 at 21 days post boost (Fig. 2a). We found that BNT162b2 induced protective immunity against SARS-CoV-2 Alpha and Omicron BA.1 variant in both WT and μMT C57BL/6 J mice. BNT162b2 vaccination significantly inhibited Alpha and Omicron BA.1 replication in the lung tissues and nasal turbinate (NT) tissues of both WT and μMT mice at 2 dpi or 4 dpi (Fig. 2b, c). Despite the lack of B cells, BNT162b2 vaccination reduced Alpha replication in the lungs of μMT mice by 11.5-fold (*P* = 0.0274) and 11.6-fold (*P* = 0.0151) on 2 and 4 dpi, respectively (Fig. 2b). Similarly, BNT162b2 vaccination reduced Omicron BA.1 replication in the lungs of μMT mice by 3.1-fold (P = ns) and 50-fold (*P* = 0.0188) on 2 and 4 dpi, respectively (Fig. 2c).

SARS-CoV-2 pathogenesis is associated with the accumulation of proinflammatory myeloid cells within the lung tissue in mouse model^16,17^. To identify correlates of vaccine-induced immunity in C57BL/6 J mice, we assessed the histopathological changes in the lung tissues of vaccinated and unvaccinated mice after SARS-CoV-2 Alpha and Omicron BA.1 challenge. For mice infected with Alpha, lungs in vaccinated WT mice had the least severe pulmonary pathology with relatively intact structure at 2 dpi or 4 dpi (Fig. 2d). More interstitial pneumonia and immune cell influx were observed in unvaccinated WT mice. Similarly, reduced in vivo pathology was detected in vaccinated μMT mice when compared with the unvaccinated ones. For mice infected with Omicron BA.1, the pathology of virus-induced lung tissues was milder than that of counterparts infected with Alpha, in keeping with recent findings^18^ (Fig. 2d, e). In addition, the degree of inflammatory cell infiltration in the lungs of vaccinated μMT mice was lower than that observed in the lungs of un-vaccinated μMT mice (Fig. 2d, e). Together, these findings indicated that BNT162b2 vaccination is capable of inducing protective immunity in μMT mice in the absence of the antibody response, suggesting cellular immunity may serve to protect against SARS-CoV-2 infection.

**Fig. 3:**
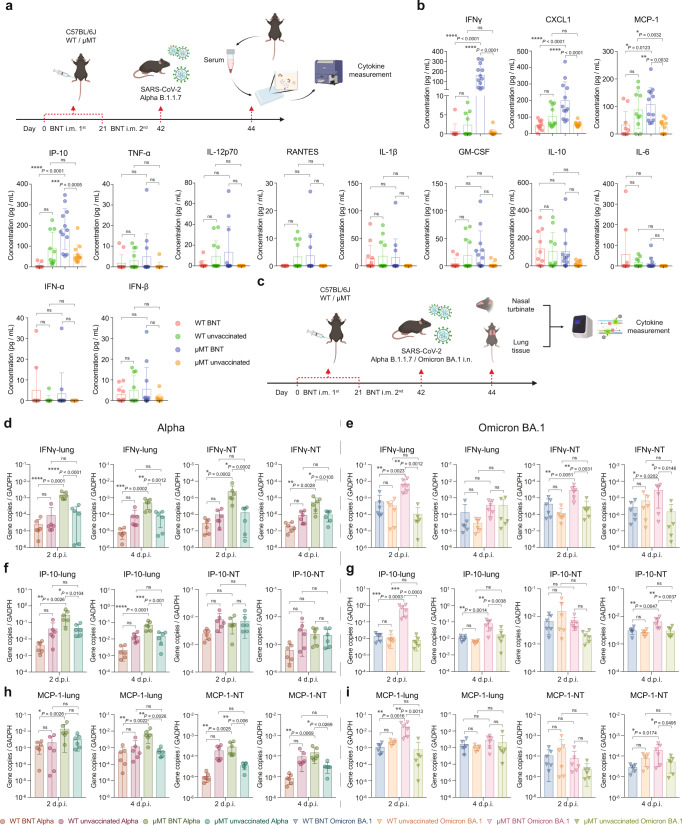
BNT162b2 stimulates substantially higher levels of cytokines/chemokines in C57BL/6 J μMT mice. **a** Schematic diagram of vaccination, viral challenge, and multiplex cytokine/chemokine profiling. **b** Cytokine/chemokine profiles in blood samples collected from BNT162b2 vaccinated and unvaccinated C57BL/6 J WT/μMT mice at 2 d.p.i. of Alpha variant determined by multiplex profiling (For WT mice, *n* = 10; μMT mice, *n* = 12). **c** Schematic diagram of vaccination, viral challenge, and cytokine/chemokine measurement by q-RTPCR. **d**–**i** The IFN-γ, IP-10, and MCP-1 expression was determined by q-RTPCR using RNA samples extracted from lung and NT from BNT162b2 vaccinated and unvaccinated C57BL/6 J WT/μMT mice at 2 d.p.i. and 4 d.p.i. of Alpha (**d**, **f**, **h**) or Omicron BA.1 variant (e, g, i) (*n* = 6). Data are presented as mean ± SD. Statistical significance was calculated using one-way ANOVA test (*p < 0.05, **p < 0.01, ***p < 0.001, ****p < 0.0001, ns = not significant). Figure 3a, c were created with BioRender.com.

Cytokines are key coordinators of the cellular immune response and essential to the activation of T cells. We next evaluated the impact of BNT162b2 vaccination on the cytokine milieu in mice infected with SARS-CoV-2 Alpha (Fig. 3a, b). Cytokine profiling of blood samples from mice challenged with Alpha revealed that IFN-γ, CXCL1, MCP-1 and IP-10 protein concentrations were significantly higher in vaccinated μMT mice than those in the unvaccinated mice (Fig. 3c). In particular, Alpha challenge drove a 253-fold (*p* < 0.0001) increase of IFN-γ secretion in vaccinated μMT mice when compared to un-vaccinated μMT mice (Fig. 3c), suggesting strong activation of the cellular immunity upon viral challenge in vaccinated μMT mice. On the contrary, BNT162b2 vaccination is associated with a marked decrease of these key cytokines in WT mice when compared with unvaccinated ones (Fig. 3c).

We next measured the cytokine levels in lung and NT tissue of vaccinated and unvaccinated mice upon Alpha or Omicron BA.1 infection by quantitative reverse transcription PCR (RT-qPCR). In line with the data in blood samples, BNT162b2 vaccination increased the expression of IFN-γ, MCP-1, and IP-10 in lung and NT tissues of μMT mice challenged with Alpha or Omicron BA.1 (Fig. 3d–i).

**Fig. 4:**
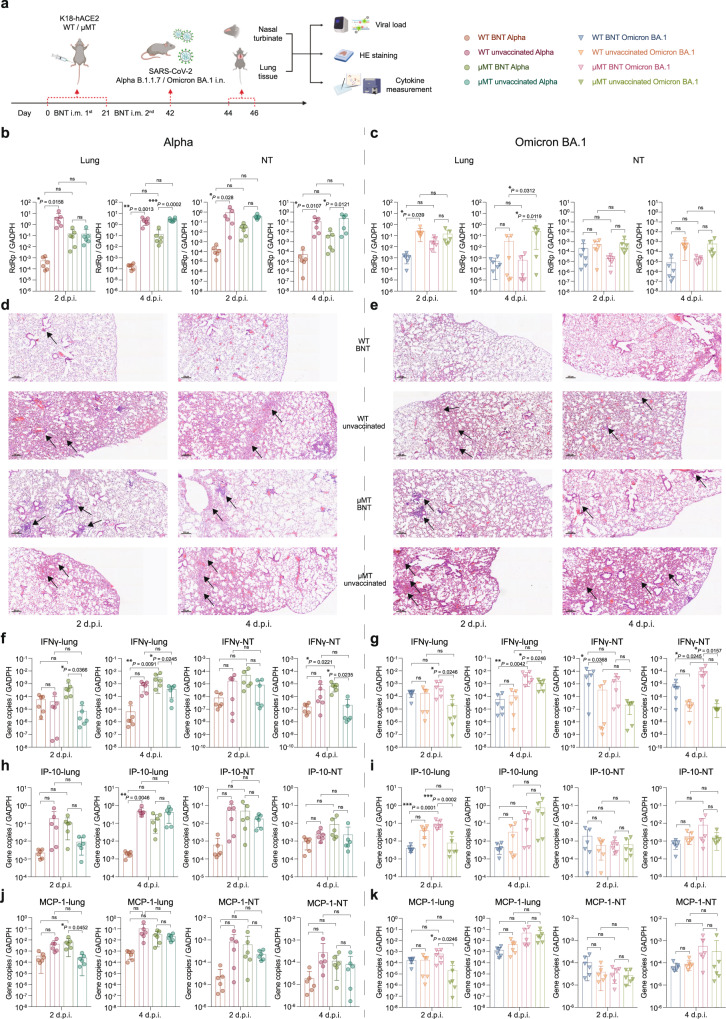
BNT162b2 also offers protection against SARS-CoV-2 in K18-hACE2 μMT mice. **a** Schematic diagram of vaccination, viral challenge and cytokine/chemokine measurement, and pathological studies in K18-hACE2 model. **b**, **c** The viral loads in the lung and NT of BNT162b2 vaccinated and unvaccinated K18-hACE2 WT/μMT mice at 2 d.p.i. and 4 d.p.i. (*n* = 6). **d**, **e** Representative images of the H&E-stained lung tissues of BNT162b2 vaccinated and unvaccinated K18-hACE2 WT/μMT mice challenged with Alpha (**d**) or Omicron BA.1 variant (**e**). **f**–**k** The IFN-γ, IP-10, and MCP-1 level was quantified by q-RTPCR using RNA samples extracted from lung and NT from BNT162b2 vaccinated and unvaccinated K18-hACE2 WT/μMT mice at 2 d.p.i. and 4 d.p.i. of Alpha (**f**, **h**, **j**) or Omicron BA.1 variant (**g**, **i**, **k**) (*n* = 6). Scale bar = 200 μm. Data are presented as mean ± SD. Statistical significance was calculated using one-way ANOVA test (*p < 0.05, **p < 0.01, ***p < 0.001, ****p < 0.0001, ns = not significant). Figure 4a was created with BioRender.com.

The K18-hACE2 mouse model has been frequently used to assess SARS-CoV-2 pathogenesis^12,19^. To evaluate the impact of B cell depletion in the K18-hACE2 mice, we obtained K18-hACE2 μMT mice by breeding K18-hACE2 mice with μMT mice. In the K18-hACE2/μMT mice, BNT162b2 vaccination similarly offered protection against Alpha and Omicron BA.1 infection. Alpha and Omicron BA.1 replication in the lung and NT tissues were lower in vaccinated K18-hACE2/μMT mice when compared with un-vaccinated ones (Fig. 4b, c). In line with the results in C57BL/6 J model, we observed ameliorated lung pathology in vaccinated WT or μMT mice when compared with unvaccinated ones (Fig. 4d, e). For cytokine profiling, we quantified IFN-γ, MCP-1, and IP-10 expression in lung tissue and NT in the mice by RT-qPCR. We found that BNT162b2 vaccination increased the expression of IFN-γ, MCP-1, and IP-10 in lung and NT tissues of K18-hACE2 μMT mice challenged with Alpha or Omicron BA.1 (Fig. 4f-k), similar to what we observed in C57BL/6 J μMT mice (Fig. 3d–i). Taken together, these findings indicate that BNT162b2 elicits significant protective immunity against SARS-CoV-2 Alpha and Omicron BA.1 in B lymphocyte-deficient mice.

### T cell-mediated immunity is highly activated in B lymphocyte-deficient mice upon SARS-CoV-2 infection

**Fig. 5:**
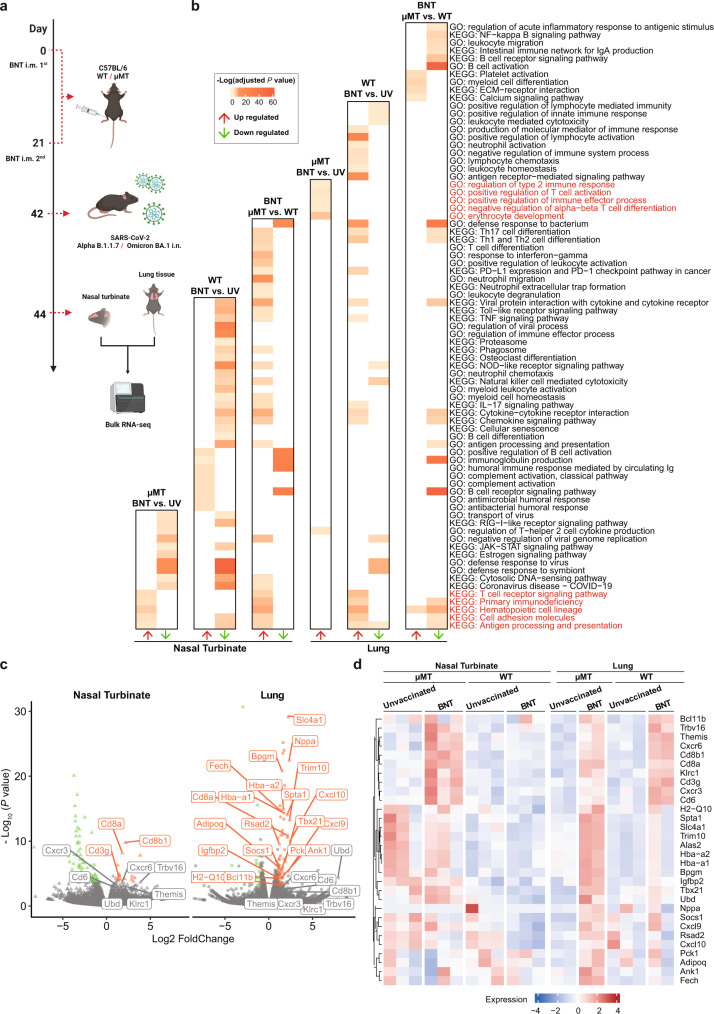
Transcriptome profiles of BNT162b2 vaccinated µMT mice after SARS-CoV-2 challenge are determined by bulk RNA-Seq. **a** Schematic diagram of the experimental design of the RNA-seq. C57BL/6 J µMT and WT mice were either unvaccinated or vaccinated with BNT162b2 three weeks before SARS-CoV-2 Alpha challenge. NT and lung tissues were then collected 48 h after the challenge for further RNA-seq library construction and sequencing. **b** Heatmap plot showing Gene ontology and KEGG pathway where significantly differentially expressed genes are enriched. The colored block indicated genes enriched terms showed in the right with corresponding Benjamini-Hochberg adjusted *P* values. (UV: unvaccinated) **c** Volcano plot showing differential expression genes from DESeq2 comparing BNT162b2 vaccinated transcriptome to unvaccinated ones in both NT and lung tissues of µMT mice. Genes with orange labels were significantly upregulated in NT or lung (Benjamini-Hochberg adjusted *P* value < 0.05 and gene expression fold change > 2), scatters with grey labels indicated gene upregulated in both NT and lung (Benjamini-Hochberg adjusted *P* value < 0.05 and gene expression fold change > 2). **d** Heatmap plot showing the expression levels of upregulated genes labeled in (**b**) across different conditions, expression of each gene was scaled in NT and lung, respectively. Figure 5a was created with BioRender.com.

To characterize the vaccine-induced protective immunity in μMT mice, we performed bulk RNA-seq of WT and µMT C57BL/6 J mice lung and NT tissues with or without vaccination on 2 dpi (Fig. 5a). Gene functional enrichment analysis based on the significantly differential expressed genes showed prominent immune characters between different conditions (Fig. 5b). We found that BNT162b2 vaccination boosted both humoral and cellular immunity in both NT and lung tissues in WT mice (Fig. 5b). In contrast, due to the lack of mature B cells, only genes involved in T cell activation and T cell receptor signaling pathways were significantly upregulated in vaccinated µMT mice comparing to unvaccinated µMT mice, suggesting that cellular immunity can be effectively activated by BNT162b2 vaccination in B cell-deficient mice (Fig. 5b). Cell type functional enrichment also indicated that the function of CD8 + T cell is highly enriched in vaccinated µMT mice when compared to the unvaccinated µMT mice (Fig. S2). According to the differential gene expression analysis, we found that T cell-related genes/pathways were significantly upregulated in the vaccinated µMT mice in both NT and lung tissue when compared to unvaccinated µMT mice (Fig. 5c, d). Among these genes, integral membrane glycoprotein CD8a serves as a coreceptor of class I MHC molecular in CD8^+^ T cells, and cell adhesion molecular CD6 that regulates T cell responses was both upregulated in NT and lung tissues (Fig. 5c, d). Besides, the *cxcr6* gene, which controls the localization of resident memory T lymphocytes to different compartments of the lung and maintains airway resident memory T lymphocytes^20^, was also significantly upregulated in vaccinated µMT mice (Fig. 5c, d).

### IFN-γ facilitates vaccine-induced immunity in B lymphocyte-deficient mice

**Fig. 6:**
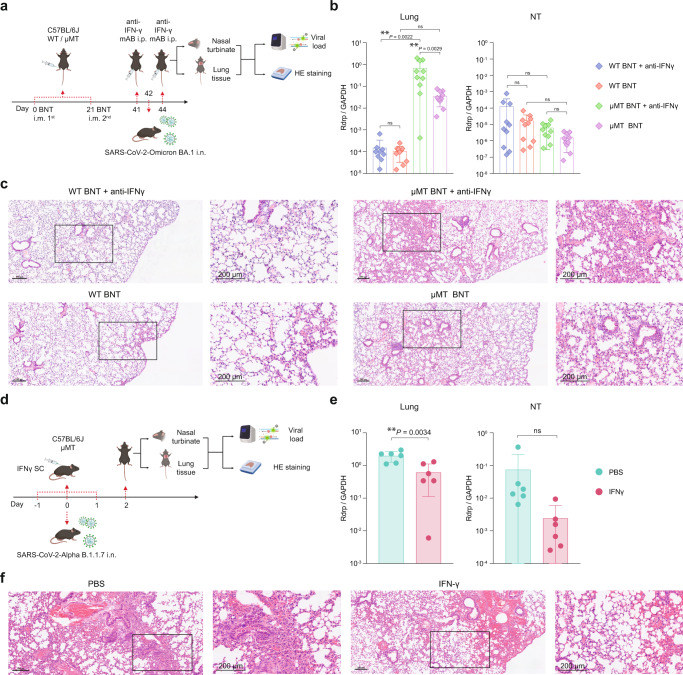
Vaccine-induced immunity in μMT mice is associated with elevated IFN-γ expression, CD4^+^ and CD8^+^ T cells. **a** Schematic diagram of vaccination, IFN-γ depletion, viral challenge, and pathological studies. **b** The viral loads in the lung and NT of BNT162b2 vaccinated and unvaccinated C57BL/6 J μMT mice at 2 d.p.i. with IFN-γ depletion (*n* = 10). (mIFN-γ, anti-IFN-γ monoclonal antibody). **c** Representative images of the H&E-stained lung tissues of BNT162b2 vaccinated WT/μMT mice treated with mIFN-γ or PBS. Scale bar = 200 μm. **d** Schematic diagram of IFN-γ administration in naïve μMT mice upon Alpha challenge. **e** The viral loads in the lung and NT of naïve μMT mice supplemented with IFN-γ or PBS at 2 d.p.i. (*n* = 6). **f** Representative images of the H&E-stained lung tissues of naive μMT mice treated with IFN-γ or PBS. Scale bar = 200 μm. Data are presented as mean ± SD. Statistical significance was calculated using one-way ANOVA test or unpaired two-tailed Student’s *t*-test. (*p < 0.05, **p < 0.01, ***p < 0.001, ****p < 0.0001, ns = not significant). Figure 6a, d were created with BioRender.com.

As mentioned earlier, IFN-γ secretion was substantially upregulated in serum, NT, and lung tissues of vaccinated μMT mice. To further evaluate the role of IFN-γ in the vaccine-induced protective immunity in μMT mice, we injected IFN-γ depleting antibody into vaccinated WT and μMT mice before Omicron BA.1 challenge (Fig. 6a). Our results demonstrated that the IFN-γ depleting antibody significantly increased Omicron BA.1 replication by 19.3-fold (*P* = 0.0029) in the lung of vaccinated μMT mice. In contrast, the IFN-γ depleting antibody did not modulate Omicron BA.1 replication in the lungs of vaccinated WT mice (0.7-fold, *P* = NS) (Fig. 6b). In the NT, the IFN-γ depleting antibody has a trend to promote virus replication in vaccinated WT and μMT mice, albeit no statistical significance was reached (Fig. 6b). In keeping with these observations, treatment of IFN-γ depleting antibody resulted in more severe lung pathology in vaccinated μMT mice while no significant change in lung pathology was observed in vaccinated WT mice when IFN-γ depleting antibody was administered (Fig. 6c). A recent study reported that supplementation of IFN-γ reverses the age-dependent COVID-19 phenotype in mice^21^. Therefore, we next treated naïve μMT mice daily with 10 μg IFN-γ from −1 to 1 d.p.i. to investigate whether administration of IFN-γ could alleviate the disease severity upon SARS-CoV-2 infection. Supplementation of IFN-γ resulted in lower viral load and milder lung pathology in NT and lung tissues of naïve μMT mice than PBS control group when infected with SARS-CoV-2 Alpha (Fig. 6e, f). Together, our results indicate that IFN-γ contributes to the vaccine-induced cellular immunity that offers protection against SARS-CoV-2 in B lymphocyte-deficient mice.

### CD4^+^ and CD8^+^ T cells are required for the vaccine-induced clearance of SARS-CoV-2 in NT and lung tissue in B lymphocyte-deficient mice

**Fig. 7:**
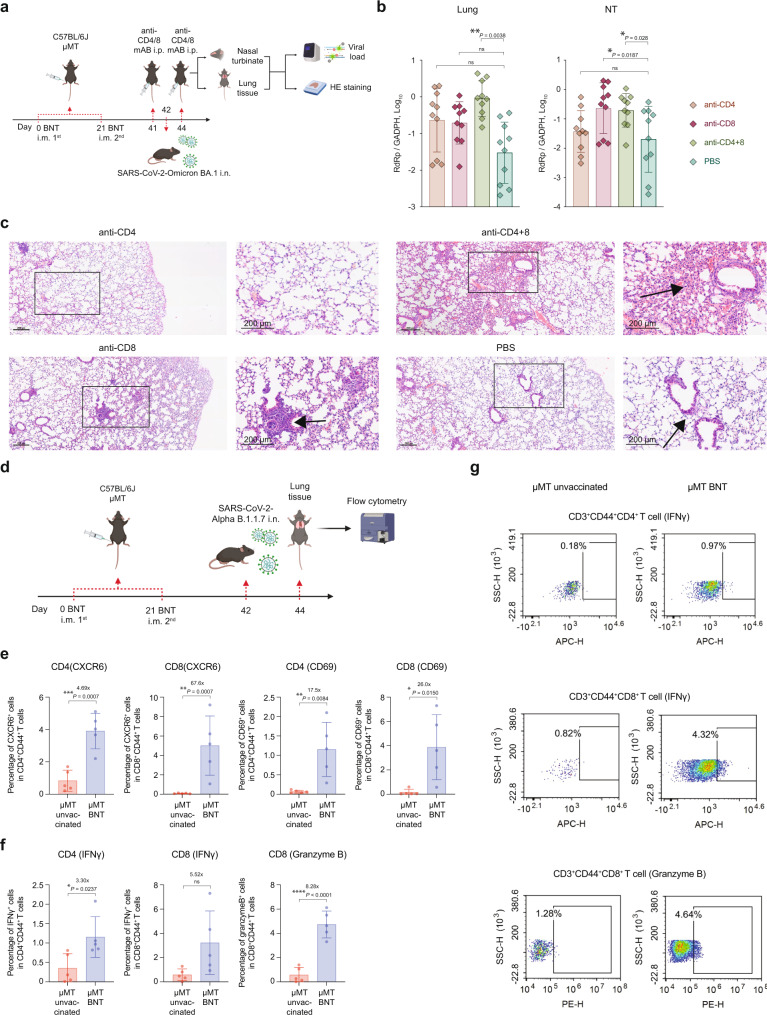
CD4^+^ and CD8^+^ T cells are required for the vaccine-induced clearance of SARS-CoV-2 in NT and lung tissue in μMT mice. **a** Schematic diagram of vaccination, CD4^+^ or CD8^+^ T cell depletion, viral challenge, and pathological studies. **b** The viral loads in the lung and NT of BNT162b2 vaccinated and unvaccinated C57BL/6 J μMT mice at 2 d.p.i. with CD4^+^, CD8^+^, or both T cell depletion (*n* = 10). (aCD4, anti-CD4 monoclonal antibody; aCD8, anti-CD8 monoclonal antibody). **c** Representative images of the H&E-stained lung tissues of BNT162b2 vaccinated μMT mice treated with aCD4, aCD8, both depleting antibodies or PBS. Scale bar = 200μm. **d** Schematic diagram of flow cytometry analysis of CD4^+^ and CD8^+^ T cells in lung tissues of vaccinated and unvaccinated C57BL/6 J μMT mice after Alpha infection. **e** Percentage of CXCR6^+^ and CD69^+^ CD4/8^+^ T cells in lung tissues of vaccinated and unvaccinated C57BL/6 J μMT mice after Alpha infection (*n* = 5). **f** Percentage of CD44^+^ IFN-γ^+^ CD4/8^+^ and CD44^+^ Granzyme B^+^ CD8^+^ T cells in lung tissues of vaccinated and unvaccinated C57BL/6 J μMT mice after Alpha infection (*n* = 5). **g** Representative dot plots showing IFN-γ- and Granzyme B-producing CD44^+^ CD4/8^+^ T cells in lung tissues of vaccinated and unvaccinated C57BL/6 J μMT mice after Alpha infection. Data are presented as mean ± SD. Statistical significance was calculated using one-way ANOVA test or unpaired two-tailed Student’s *t*-test (*p < 0.05, **p < 0.01, ***p < 0.001, ****p < 0.0001, ns = not significant). Figure 7a, d were created with BioRender.com.

As previously mentioned, CD4^+^ and CD8^+^ T memory cells in vaccinated μMT mice produce IFN-γ upon spike protein stimulation (Fig. 1c, d). We then investigated if CD4^+^ and CD8^+^ T cells contribute to the vaccine-induced cellular immunity in μMT mice by using CD4-, CD8-, or CD4/CD8-depleting antibodies. In lung tissues, CD4-, CD8-, and CD4/CD8-depleting antibodies increased Omicron BA.1 replication by 10-fold (*P* = NS), 10-fold (*P* = NS), and 42-fold (*P* = 0.0038), respectively (Fig. 7b). In the NT tissues, CD4-, CD8-, and CD4/CD8-depleting antibodies increased Omicron BA.1 replication by 0.6-fold (*P* = NS), 15.6-fold (*P* = NS), and 7.6-fold (*P* = 0.028), respectively (Fig. 7b). In keeping with the virus replication findings, simultaneous depletion of both CD4^+^ and CD8^+^ T cells resulted in the most severe lung pathology in vaccinated μMT mice among all evaluated groups (Fig. 7c). To further dissect the role of CD4^+^ and CD8^+^ T cells, we characterized tissue-resident memory (TRM) and effector cells in lung tissues of vaccinated μMT mice by flow cytometry analysis. Consistent with the bulk RNA-Seq data, our results demonstrated that 26-fold (*P* = 0.015) higher percentage of CD44^+^ CD69^+^ CD8^+^ T cells were found in lung tissues of vaccinated μMT mice than in unvaccinated ones after SARS-CoV-2 Alpha infection (Fig. 7e). In addition, the percentage of CXCR6^+^ lung tissue-resident memory (TRM) CD8^+^ T cells was also significantly higher (67.6-fold, *P* = 0.0007) in vaccinated μMT mice (Fig. 7e, S7a). More IFN-γ^+^ and Granzyme B^+^ CD8^+^ T cells were detected in vaccinated μMT mice (5.5-fold, *P* = 0.0159 and 8.3-fold, *p* < 0.0001, respectively) when compared with unvaccinated μMT mice, suggesting that robust cellular immunity was induced in vaccinated μMT mice upon SARS-CoV-2 challenge (Fig. 7f, g). Likewise, the percentage of CD44^+^ CD69^+^ CD4^+^, CXCR6^+^ CD4^+^, and IFN-γ^+^ CD4^+^ T cells were also higher in vaccinated μMT mice than unvaccinated mice, but less significant differences were found (Fig. 7e–g, S7a, b). Taken together, these data indicate that while both CD4^+^ and CD8^+^ T cells are required for optimal vaccine-induced protection against SARS-CoV-2 in lung tissues of μMT mice, CD8^+^ T cells play a more predominant role than CD4^+^ T cells in the NT of μMT mice.

### BNT162b2 induces protective immunity against SARS-CoV-2 Omicron BA.5.2 in B lymphocyte-deficient mice

**Fig. 8:**
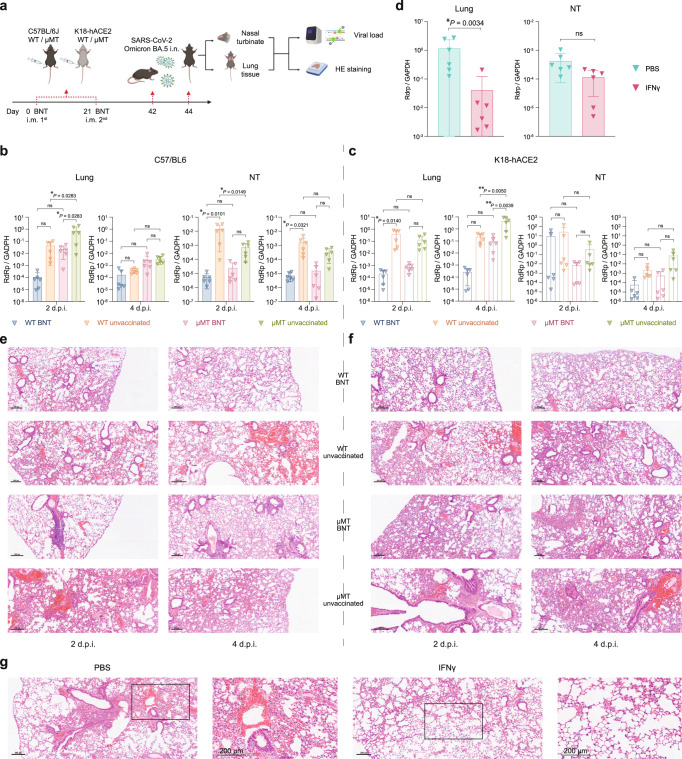
BNT162b2 induces protective immunity in C57BL/6 J and K18-hACE2 μMT mice against Omicron BA.5.2. **a** Schematic diagram of vaccination, viral challenge, and pathological studies in C57BL/6 J and K18-hACE2 model. **b**, **c** The viral loads in the lung and NT of BNT162b2 vaccinated/unvaccinated C57BL/6 J and K18-hACE2 WT/μMT mice at 2 d.p.i. and 4 d.p.i. (*n* = 6). **d** The viral loads in the lung and NT of naïve μMT mice supplemented with IFN-γ or PBS at 2 d.p.i. upon Omicron BA.5.2 infection (*n* = 6). **e**, **f** Representative images of the H&E-stained lung tissues of BNT162b2 vaccinated/unvaccinated C57BL/6 J (**e**) and K18-hACE2 (**f**) WT/μMT mice challenged with Omicron BA.5.2 Scale bar =  200  μm. **g** Representative images of the H&E-stained lung tissues of naive μMT mice treated with IFN-γ or PBS. Scale bar = 200 μm. Data are presented as mean ± SD. Statistical significance was calculated using one-way ANOVA test (*p < 0.05, **p < 0.01, ***p < 0.001, ****p < 0.0001, ns = not significant). Figure 8a was created with BioRender.com.

SARS-CoV-2 Omicron BA.5, which has substantially higher immune escape capacity than BA.1, are the dominant strain in many countries as of January 2023^22–24^. To test whether BNT162b2 could induce protective immunity against SARS-CoV-2 Omicron BA.5.2 in μMT mice, we infected vaccinated C57BL/6 J and K18-hACE2 mice with SARS-CoV-2 Omicron BA.5.2 and evaluated the viral replication in these mice. The quantitative RT-PCR showed that the viral load in lung tissues of vaccinated μMT mice is 34-fold (*P* = 0.0283) and 188-fold (*P* = NS) lower than C57BL/6 J and K18-hACE2 unvaccinated μMT mice at 2 d.p.i., respectively (Fig. 8b). Although much lower viral load was detected in NT of both C57BL/6 J and K18-hACE2 vaccinated mice at 2 and 4 d.p.i., the statistical difference was not seen between the vaccinated and unvaccinated groups (Fig. 8b). In line with the viral replication, the lung pathology was also alleviated in vaccinated mice (Fig. 8c). Consistent with the therapeutic effect of IFN-γ in naïve μMT mice against SARS-CoV-2 Alpha, supplementation of IFN-γ could also mitigate the viral replication and lung pathology in naïve μMT mice upon Omicron BA.5.2 challenge (Fig. 8d, e).

## Discussion

A large body of evidence has emphasized the essential role of neutralizing antibodies induced by vaccines or natural infections in the protection against SARS-CoV-2. BNT162b2 vaccine has been reported to elicit an adaptive humoral and poly-specific cellular immune response in both animal models and humans^1–3^. In this study, we investigated whether vaccine-induced cellular immunity is sufficient to elicit protective immunity against SARS-CoV-2 by using µMT mice that lack mature B cells. A recent study has demonstrated that both humoral and cellular adaptive immunity are important in viral clearance after primary infection of SARS-CoV-2 by using AAV-hACE2 µMT mice^25^. Here, we describe the use of two different mouse models to identify components of adaptive immunity responsible for viral clearance induced by BNT162b2.

We measured the viral load in both nasal turbinate (NT) and lung tissue of the vaccinated and unvaccinated mice after the viral challenge. The clearance of SARS-CoV-2 is dramatically more effective in both vaccinated C57BL/6 J WT and µMT mice when compared with unvaccinated ones, suggesting the induction of sufficient cellular immunity by BNT162b2 in mice without humoral responses. Similar results were obtained by using K-18 hACE2 mouse model, though a quicker clearance of SARS-CoV-2 was observed in C57BL/6 J mice as indicated in the previous study^11^. For survival test in K18-hACE2 model, vaccination of BNT162b2 resulted in a 20% survival rate in µMT mice as opposed to 0% in unvaccinated µMT mice in the setting of Alpha variant challenge (Fig. S3a). Interestingly, a substantially higher level of viral RNA was detected in the brain tissue of vaccinated µMT mice when compared with that in vaccinated WT mice, suggesting antigen-specific antibodies are essential to contain the viral replication in brain tissue (Fig. S3c). When vaccinated µMT mice were challenged with Omicron BA.1, no death was caused due to the attenuated virulence of Omicron BA.1 (Fig. S3b)^18^. Bulk RNA-seq analysis revealed that pathways involved in T cell activation were upregulated in vaccinated µMT mice when compared to unvaccinated ones. Moreover, we found that the gene functional enrichment profile of vaccinated WT vs. µMT mice in nasal turbinate was different from what is in lung tissue. In nasal turbinate, T cell differentiation and receptor signaling pathways are upregulated in vaccinated µMT mice. Nevertheless, SARS-CoV-2 infection induced upregulated pathways involved in hemostasis and thrombosis (platelet activation, ECM-receptor interaction, etc.) in vaccinated µMT mice in lung tissue when compared to WT mice (Fig. S4), implying that the mechanism of vaccine-induced viral clearance in the lung might be different from that in nasal turbinate in µMT mice. It is also important to point out that antibodies can also function by opsonization and complement-mediated cytotoxicity. To investigate the role of the complement system in the clearance of SARS-CoV-2, we carried out gene set enrichment analysis (GSEA) on the gene sets related to complement pattern recognition, complement proteases, complement component, complement receptor, and complement regulator. We found that the complement activation pathways were upregulated in vaccinated WT mice when compared with unvaccinated WT mice (Fig. 5b). No significant differences in the complement-related pathways were detected in either NT or lung tissues between vaccinated and unvaccinated μMT mice (Fig. S5).

To confirm whether T cell-mediated immunity contributes to the vaccine-induced protective immunity in µMT mice, we analyzed the cytokine/chemokine profile of the blood, NT, and lung samples from vaccinated WT and µMT mice. We demonstrated that IFN-γ secretion is highly activated in vaccinated µMT mice upon viral challenge. Particularly, a 253-fold increase of IFN-γ secretion was observed in vaccinated μMT mice compared to vaccinated WT mice. Likewise, CXCL10 (IP-10) secretion in response to IFN-γ was also significantly higher in vaccinated μMT mice. These data are consistent with our finding that stimulation of spike protein of SARS-CoV-2 induces a higher percentage of IFN-γ-secreting CD4^+^ and CD8^+^ memory T cells in vaccinated µMT mice (Fig. 1c, d). By using depleting antibodies against IFN-γ, we confirmed that IFN-γ plays an important role in viral clearance in vaccinated μMT mice but not WT mice. Upon viral infection, IFN-γ acts directly on CD8^+^ T cells to facilitate their differentiation into cytotoxic T lymphocytes, which secret cytokines to restrain viral replication and clear infected cells^26^. The role of IFN-γ during the infection of SARS-CoV-2 is not yet fully understood^27^. Previous studies showed that IFN-γ was present in high amounts in patients with moderate to severe COVID-19 along with other proinflammatory cytokines and was associated with cytokine storm and acute lung damage in COVID-19 patients^28,29^. In this study, we demonstrated that IFN-γ facilitated the clearance of SARS-CoV-2 without inducing lethal cytokine storm in vaccinated µMT mice. Increased IFN-γ levels in lungs of vaccinated µMT mice did not either result in more severe lung pathology. Moreover, the levels of many proinflammatory cytokines such as IL-6, IL-1β, TNF-α, and other interferons were not significantly elevated in vaccinated µMT mice. On the other hand, a recent study reported that therapeutic administration of IFN-γ could reverse the disease severity upon SARS-CoV-2 infection in aged WT C57BL/6 mice^21^. In line, we showed that supplementation of IFN-γ was effective in lowering the viral load in NT and lung tissues of naïve µMT mice, confirming the pivotal role of IFN-γ in vaccine-induced viral clearance in µMT mice. However, the administration of IFN-γ in naïve μMT mice resulted in less significant reduction of viral load (3.3-fold for Alpha and 22-fold for BA.5.2, respectively) when compared with the BNT vaccination (11.5-fold for Alpha and 33.8-fold for BA.5.2, respectively). This implies that element(s) other than IFN-γ might be involved in the vaccine-induced protection. Together, we hypothesize that BNT162b2 induced IFN-γ-mediated compensatory protective immunity in the absence of humoral immunity in µMT mice without causing any detrimental effect of IFN-γ.

CD4^+^ and CD8^+^ T cells are the major sources of IFN-γ production in the adaptive immune system^30^. We showed that depletion of CD8^+^ T cells in vaccinated μMT mice lead to a 15.6-fold increase of viral load in NT compared to PBS group, which is in line with the highly enriched function of CD8 + T cell in vaccinated µMT mice by cell type enrichment analysis from RNA-seq data (Fig. S2). However, only the depletion of both CD4^+^ T cells and CD8^+^ T cells resulted in a significant increase of the viral RNA load in NT or lung tissue from vaccinated µMT mice. Therefore, our data indicate that both CD4^+^ and CD8^+^ T cells play important roles in the vaccine-induced clearance of SARS-CoV-2 in B cell-deficient mice. By using flow cytometry, we demonstrated that a significantly higher percentage of CD44^+^ CD69^+^ CD8^+^ and CXCR6^+^ lung tissue-resident memory (TRM) CD8^+^ T cells were found in lung tissues of vaccinated μMT mice than in unvaccinated ones upon SARS-CoV-2 challenge (Fig. 7e, S7a). It has been demonstrated that CD8^+^ T cells in the draining lymph nodes are one of the major producers of the circulating IFN-γ in mice after BNT162b2 vaccination^31^. Likewise, our data indicated that many more IFN-γ producing CXCR6^+^ CD8^+^ TRM cells were detected in the lung tissues of vaccinated μMT mice than unvaccinated mice upon SARS-CoV-2 infection (Fig. 7f, S7b). Taken together, we demonstrated that BNT162b2 vaccination could mount robust cellular immunity through IFN-γ-secreting T memory cells in µMT mice, promoting viral reduction. These results are consistent with the recent report that robust production of IFN-γ was observed following stimulation with spike peptide in individuals with X-linked agammaglobulinemia (XLA), who received two doses of BNT162b2^32,33^. Although XLA occurs in approximately 1 in 200,000 newborns, individuals with primary B-cell immunodeficiency (PID, 500,000 cases in the United States as of 2003, and about 50,000 cases are diagnosed each year^34^) share the symptoms with XLA individuals. Therefore, this study will shed light on the protective efficacy of BNT162b2 in these immunocompromised individuals.

SARS-CoV-2 variants such as Omicron variants that significantly evade humoral immunity are continuously emerging. Here, we demonstrated that vaccination of BNT162b2 elicited protective immunity against Omicron BA.1 and BA.5.2 in either WT or μMT mice, although the efficacy is less significant than that against Alpha. These results are consistent with the observation that current vaccines still show robust protection against severe disease caused by Omicron and its sub-lineages despite the significantly decreased neutralizing antibody responses in the human population^35^. The protection was found to be strongly associated with vaccine-induced cellular immunity that is mediated by CD8^+^ T cells. Likewise, we showed that spike protein of Omicron BA.1 could stimulate the robust secretion of IFN-γ by CD8^+^ T cells in both WT and µMT mice vaccinated with BNT162b2 (Fig. 1c-d).

Current COVID-19 vaccines elicit robust systemic immunity but poor immunity at the respiratory mucosa^36^. With enhanced immune evasion of the emerging Omicron variants and waning systemic immunity among the population, these vaccines such as BNT162b2 are less effective at containing viral transmission. Successful vaccine-induced mucosal immune memory within the respiratory tract is the key to preventing viral transmission. However, we did not investigate the role of vaccine-induced mucosal T cell immunity in B-cell-deficient mice in this study. Mao et al.^37^ recently developed a vaccine strategy that elicited protective mucosal immunity against SARS-CoV-2 and reduced viral transmission in K18-hACE2 mice. Hence, it would be worthwhile to study if mucosal T cell immunity could reduce viral transmission in immunocompromised mouse models under the improved vaccine strategy.

A growing body of publications has reported that robust Spike-specific T cell responses were induced in BNT162b2 vaccinated patients with impaired humoral responses^4–6^. In this study, our data from mouse models are consistent with the above-mentioned reports in humans, demonstrating the validity of our animal model to evaluate the COVID-19 vaccine efficacy in the context of impaired humoral immunity.

We further demonstrated that T cell-mediated immunity provides significant immunity in the absence of antibody-mediated immunity in vaccinated μMT mice upon SARS-CoV-2 infection and retains the ability to respond to emerging variants of concern of SARS-CoV-2, which provide valuable insights into the clinical guidance in the vulnerable population.

## Methods

### Mice

This experimental protocol was approved by the Committee on the Use of Live Animals in Teaching and Research of the University of Hong Kong under CULATR 5479-20 and complied with the Guide for the Care and Use of Laboratory Animals published by the National Institute of Health (8^th^ edition, 2011). Six to 12-week-old mixed sex C57Bl/6 J (WT), B6.129S2-Ighmtm1Cgn/J (#002288, μMT), and B6.Cg-Tg(K18-ACE2)2Prlmn/J (#034860, K18-hACE2) were purchased from the Jackson Laboratory and were subsequently bred and housed at the SPF animal facility in the Center for Comparative Medicine Research (CCMR) of the University of Hong Kong. To obtain K18-hACE2 μMT mice, B6.Cg-Tg(K18-ACE2)2Prlmn/J mice were cross-bred with B6.129S2-Ighmtm1Cgn/J (μMT) mice. The genotyping was conducted following protocols provided by Jackson Laboratory. Mice were euthanized by overdosing with Pentobarbital sodium (250 mg/kg) intraperitoneally.

### Cell lines and viruses

Vero E6 kidney epithelial cells were cultured in Dulbecco’s modified Eagle’s medium (DMEM) supplemented with 1% sodium pyruvate and 5% fetal bovine serum (FBS) at 37 °C and 5% CO_2_. 293 T cells were cultured in DMEM supplemented with 1% sodium pyruvate and 10% FBS at 37 °C and 5% CO_2_. The cell line was obtained from the American Type Culture Collection (ATCC) and has been tested negative for contamination with mycoplasma. SARS-CoV-2 isolates used in the study were previously described^18,38^ and were amplified in VeroE6 cells overexpressing transmembrane serine protease 2 (TMPRSS2). The VeroE6-TMPRSS2 cells were infected at a multiplicity of infection of 0.01 for 2 to 3 days to generate a working stock, and after incubation, the supernatant was clarified by centrifugation (500 *g* × 5 min) and filtered through a 0.45-μm filter. To concentrate virus, filtered supernatants were applied to Amicon Ultra-15 centrifugal filter (Ultracel 100k) and spun at 1000 g for 15 min. The supernatant was then aliquoted for storage at −80 °C. Viral titers were measured by standard plaque assay using Vero E6 TMPRSS2 cells. In vivo and in vitro experiments with infectious SARS-CoV-2 were performed according to the approved standard operating procedures of the Biosafety Level 3 facility at the Department of Microbiology, HKU.

### Vaccination

Unused vials of Pfizer/BioNTec BNT162b2 mRNA vaccine were acquired from the Department of Health, HKSAR. Mice were anaesthetized using a mixture of ketamine (50 mg kg−1) and xylazine (5 mg kg−1), and 10 μL (1 mg) of the undiluted vaccine was injected into the left quadriceps muscle with a 32-gauge syringe.

### SARS-CoV-2–specific antibody measurements

Mouse serum samples were incubated at room temperature for 30 min before use to reduce risk from any potential virus in serum. The 96-well MaxiSorp plates (Thermo Fisher Scientific, #442404) were coated with 100 μL per well of recombinant SARS-CoV-2 spike protein at a concentration of 2 μg/ml in PBS and were incubated overnight at 4 °C. The coating buffer was removed, and plates were incubated for 1 hour at room temperature with 200 μL of blocking solution (TBS-T and 5% milk powder). Serum was diluted in dilution solution (TBS-T and 1% milk powder), and 100 μL of diluted serum was added for 2 h at room temperature. Plates were washed five times with TBS-T and 50 μL of horseradish peroxidase anti-mouse IgG (1:3000) diluted in dilution solution added to each well. After 1 h of incubation at room temperature, plates were washed five times with TBS-T. Plates were developed with 100 μL of TMB substrate solution (BD Biosciences, #555214), and the reaction was stopped after 15 min by the addition of 2 N of sulfuric acid. Plates were then read at a wavelength of 450.

### Flow cytometry

Splenocytes were isolated 21 days after the second immunization, plated at 2 × 10^6^ cells per well in 24-well plates in the complete medium [RPMI 1640 (Gibco) supplemented with 25 mM HEPES (Gibco), heat-inactivated 10% (vol/vol) FBS, low endotoxin (HyClone), and penicillin/streptomycin (Gibco)], containing spike protein trimer (50 μg/mL) at 37 °C for 16–18 h in the presence of Brefeldin A (5 μg/mL) for the last 4 h. PBS was used as a negative control. The cells were then stained with anti-CD3 PerCP-Cy5.5 (1:200, BioLegend 100218), anti-CD4 FITC (1:200, BioLegend 100406), anti-CD8 BV421 (1:200, BioLegend 100738), anti-CD44 PE-Cy7 (1:200, BioLegend 103030), fixed, permeabilized with Cytofix/Cytoperm (BD Biosciences), washed in Perm/Wash buffer (BD Biosciences), and then stained with anti–IFN-γ APC (1:200, BioLegend 505810), anti–IL-4 PE (1:200, BioLegend 504104), in Perm/Wash buffer (BD Biosciences) for 20 min at room temperature, washed twice in Perm/Wash buffer and suspended in PBS. Samples were acquired on an ACEA NovoCyte Quanteon flow cytometer (Agilent) and analyzed using NovoExpress® Software. For CD8^+^ cell differentiation study, the cells were stained with anti-CD3 PerCP-Cy5.5 (1:200, BioLegend 100218), anti-CD8 BV421 (1:200, BioLegend 100738), anti-CD44 PE-Cy7 (1:200, BioLegend 103030), anti-CD127 APC (1:200, BioLegend 135012) and anti-KLRG1 PE (1:200, BioLegend 138408).

For lung tissues harvested after viral infection, the single-cell suspension was obtained by digesting the samples with Collagenase/Hyaluronidase (StemCell) and DNase I (StemCell) solution. The samples were then stained with anti-CD3 PerCP-Cy5.5 (1:200, BioLegend 100218), anti-CD4 FITC (1:200, BioLegend 100406), anti-CD8 BV421 (1:200, BioLegend 100738), anti-CD44 PE-Cy7 (1:200, BioLegend 103030), anti-CD69 BV605 (1:200, BioLegend 104530) and anti-CXCR6 BV711 (1:200, BioLegend 151111), followed by fixation of 12 h. The cells were subsequently permeabilized with Cytofix/Cytoperm (BD Biosciences), washed in Perm/Wash buffer (BD Biosciences), and then stained with anti–IFN-γ APC (1:200, BioLegend 505810) and anti–Granzyme B PE (1:200, BioLegend 372208) in Perm/Wash buffer (BD Biosciences) for 20 min at room temperature, washed twice in Perm/Wash buffer, and suspended in PBS.

### In vivo virus challenge in mice

The use of animals was approved by the Committee on the Use of Live Animals in Teaching and Research of The University of Hong Kong under CULATR 5479-20. Experiments were repeated to give a sample size of 6 or above. Gender- and age-matched mice were randomized into different experimental groups. The mice were housed in cages with individual ventilation under 65% humidity, an ambient temperature of 21–23 °C and a 12 h–12 h day–night cycle. For virus challenge in mice, female C57BL/6 J mice (aged 6–8 weeks) or female and male K18-hACE2 transgenic mice (aged 6–8 weeks) were anaesthetized with ketamine and xylazine, followed by intranasal inoculation with 20 μl per mouse of Alpha or Omicron variants at 2 × 10^3^ p.f.u. per mouse (for K18-hACE2 transgenic mice) or 1 × 10^5^ p.f.u. per mouse (for C57BL/6 J mice) as we previously described^11,18^. Mice were euthanized by overdosing with Pentobarbital sodium at 2 and 4 d.p.i. for collecting nasal turbinate and lung tissues for virological assessment, proinflammatory cytokine quantification or histological examination. The survival of the infected animals was monitored for 14 days or until death of the animal.

### Viral RNA analysis

At indicated time points, mice were euthanized in 100% isoflurane. About 50% of total lung was placed in a bead homogenizer tube with 1 ml of PBS + 2% FBS + 2% antibiotic/antimycotic (Gibco). After homogenization, 250 μL of this mixture was placed in 750 μL of TRIzol LS (Invitrogen), and RNA was extracted with a RNeasy mini kit (Qiagen) per manufacturer protocol. To quantify SARS-CoV-2 RNA levels, we used the Luna Universal Probe One-step RT-qPCR kit (New England Biolabs) with 1 μg of RNA, using the primer/probe sets for RNA-dependent RNA polymerase (*RdRp*). The primer and probe sequences are available on request.

### Histology staining

Animal tissues were collected and fixed with 10% neutral-buffered formalin. For H&E staining, tissue sections were stained with Gill’s haematoxylin and eosin-Y. Images were acquired using Vectra Polaris automated quantitative pathology imaging system (Perkin Elmer). Three to four mice were sampled in each group and four to six sections from each animal were used for histology analysis.

### Cytokine/chemokine quantification

Cytokines/chemokines in blood samples harvested 2 d.p.i. were assessed by using LEGENDplex™ anti-virus response panel kit (BioLegend) according to the manufacturer’s instructions and quantified by using ACEA NovoCyte Quanteon flow cytometer (Agilent). The data were analyzed by LEGENDplex™ Data Analysis Software Suite. Cytokine/chemokine levels in lung or NT samples were measured by RT-qPCR with primer sets for murine IFN-γ, MCP-1 and IP-10.

### RNA-seq data processing

Adapters and low-quality bases were trimmed from raw next generation sequencing data with the help of fastp (v0.20.1) using default parameters^39^. Clean data were then used for mouse and SARS-CoV-2 gene expression quantifications using Salmon (v1.6.0) respectively^40^. Mouse gene expression profile was calculated from clean data directly. SARS-CoV-2 gene expression profile was calculated from un-mapped reads which were retrieved after executing alignment to mouse reference genome by STAR (v2.7.10a)^41^. Gene different expressions analysis were carried out by DESeq2 between different condition of the same tissue. ClusterProfiler (v3.18.1) was used for immune and viral process related gene ontologies (ontologies belongs to GO:0016032 and GO:0002376) and KEGG pathway enrichment analysis, similar GO terms were merged by “simplify” function with cutoff of 0.5^42^. Cell type functional enrichment were conducted by xCell (v1.1.0) using moue gene expression profile^43^. For Gene set enrichment analysis of the complement system, gene list related to complement system were obtained from Neha et al.^44^. Ranked fold change of genes were used during the GSEA analysis with the help of R package fgsea (v1.16.0)^45^ with default parameter.

### CD4+ and CD8+ T cell depletion

Indicated mice were injected intraperitoneally with PBS or 250 μg in 200 μL of diluted in PBS of either anti-mouse CD4 (BioXcell InVivoMab Clone GK 1.5), anti-mouse CD8 (BioXcell InVivoMab Clone 2.43), or both at indicated time points.

### IFN-γ depletion

Indicated mice were injected intraperitoneally with PBS or 250 μg in 200 μL of diluted in PBS of anti-mouse IFN-γ (BioXcell InVivoMab Clone XMG1.2) at indicated time points.

### Statistical analysis

Statistical comparisons between two experimental groups were performed using unpaired two-tailed Student’s t-tests. Comparisons among three or more experimental groups were performed using one-way or two-way ANOVA with Tukey’s multiple-comparison test. AUC values were calculated and analyzed using one-way ANOVA. The survival of animals was compared using the log-rank (Mantel–Cox) test. Differences were considered statistically significant when *P*  <  0.05. Data analysis was performed using GraphPad Prism v.8.0.

### Reporting summary

Further information on research design is available in the Nature Portfolio Reporting Summary linked to this article.

## Supplementary information

Supplementary Information

Reporting Summary

## Data Availability

Source data are provided with this paper in Source Data file. Raw bulk RNA-seq data reported in this paper have been deposited in the NCBI Sequence Read Archive under BioProject accession number PRJNA901615. Source data are provided with this paper.

## Source data

Source Data
