# Supplementary material for: Vaccine-induced protection against SARS-CoV-2 requires IFN-γ-driven cellular immune response
Source: Nat Commun. 2023 Jun 10;14:3440. doi: 10.1038/s41467-023-39096-y (PMC10257169; doi:10.1038/s41467-023-39096-y)
Supplement: Supplementary file 1 — Supplementary Information [file 41467_2023_39096_MOESM1_ESM.pdf]

Supplementary information

Vaccine-induced protection against SARS-CoV-2 requires IFN-γ-driven cellular immune response

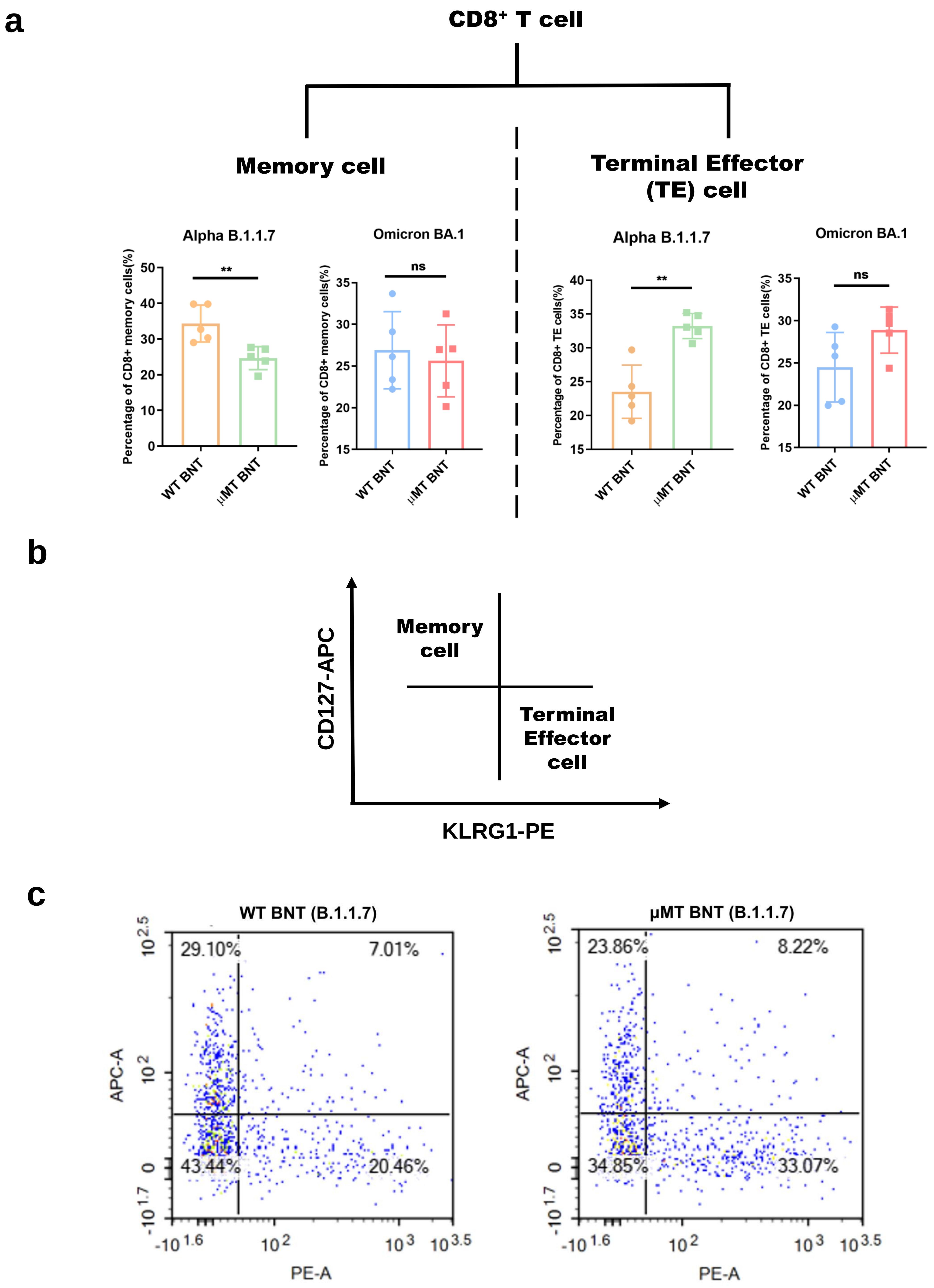

Fig. S1 Spike protein of SARS-CoV-2 induced the differentiation of CD8⁺ T memory cell in spleen of vaccinated WT and μMT mice in vitro. (a) The percentage of CD8+ T cell subsets in spleen of vaccinated WT and μMT mice after spike protein stimulation (Alpha and Omicron BA.1). (n=5) (b) Schematic diagram of characterization of CD8+ T memory cell subsets by flow cytometry. $CD127^-$ $KLRG1^+$ — TE cell; $CD127^+$ $KLRG1^-$ — memory cell. (c) Representative dot plots showing $CD8^+$ T memory cell differentiation in spleen of vaccinated WT and μMT mice. Data are presented as mean ± SD. Statistical significance was determined using unpaired two-tailed Student's t-tests.

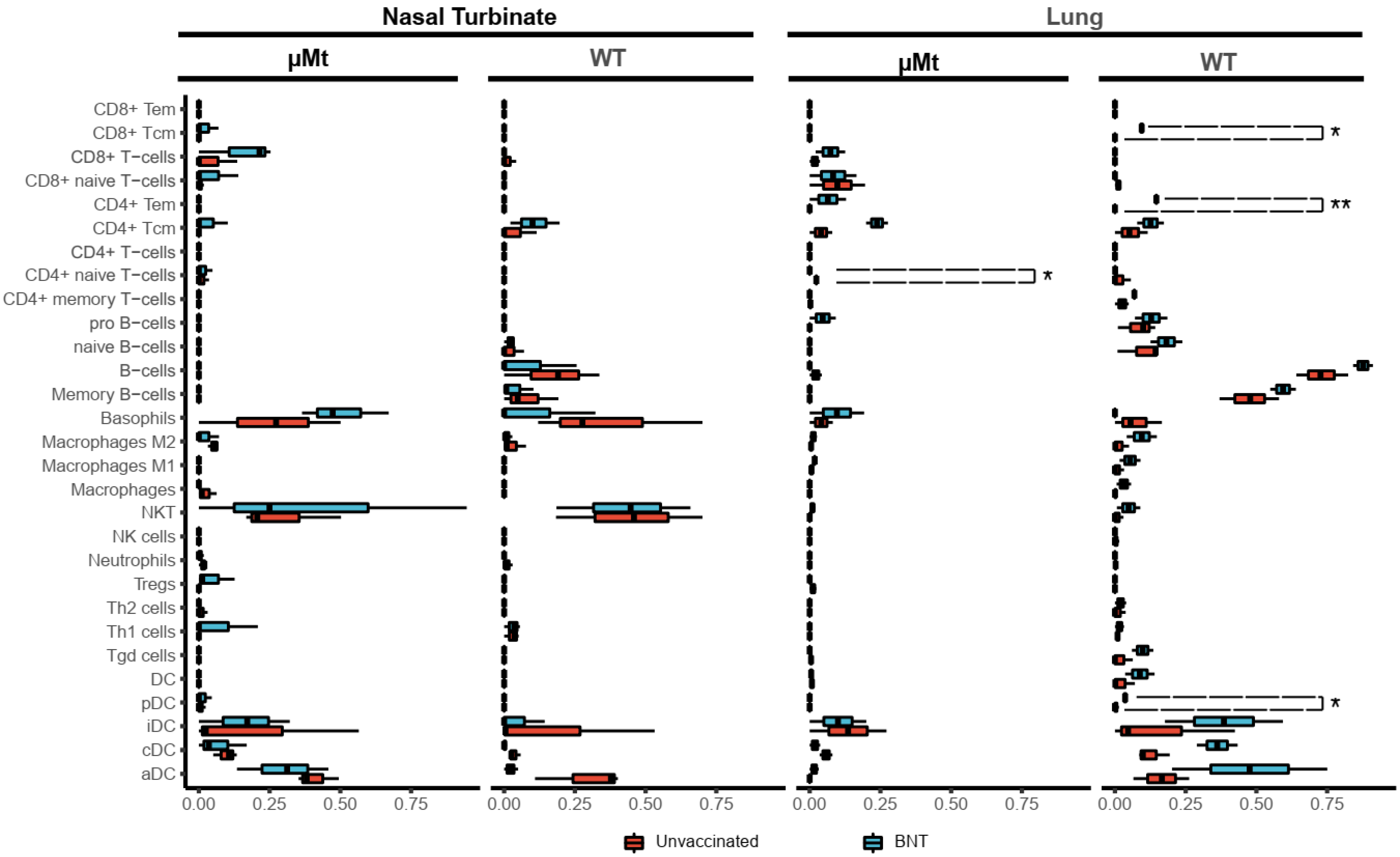

Fig. S2 Cell type enrichment analysis of the RNA-seq data. Cell type enrichment analysis of RNA-seq data indicated the function enrich scores of each cell type for vaccinated and unvaccinated WT and μMT mice. Statistical significance was determined using unpaired two-tailed Student's t-tests (*p<0.05, **p<0.01).

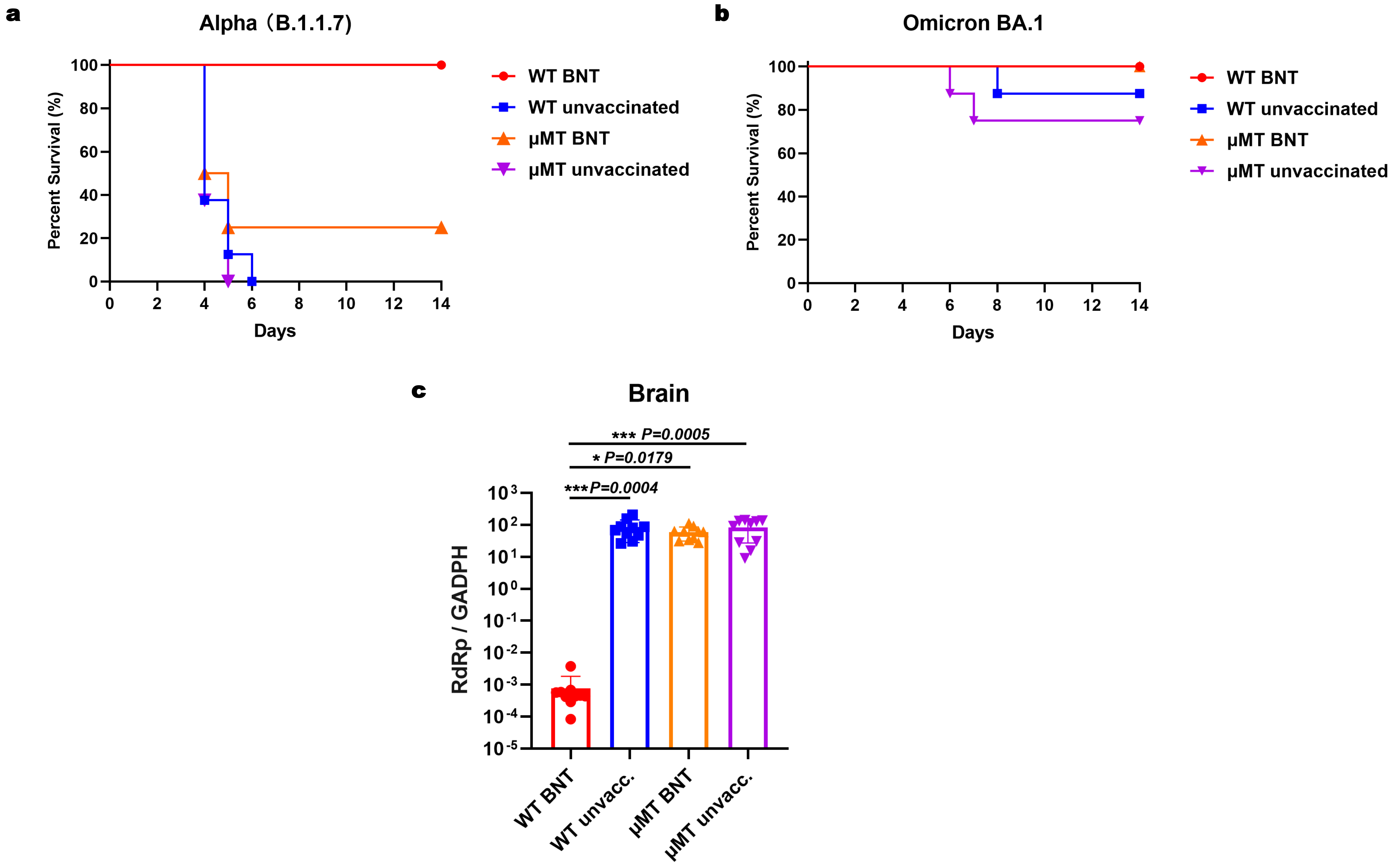

Fig. S3 BNT162b2 vaccination increased the survival of K18-hACE2 WT and μMT mice. The survival of vaccinated and unvaccinated WT and μMT mice against Alpha (a) and Omicron BA.1 (b) challenge. (n=8). Statistical significance was determined using log-rank (Mantel–Cox) tests. (c) The viral loads in the brain tissues of BNT162b2 vaccinated and unvaccinated K18-hACE2 WT/μMT mice at 3 d.p.i. (n=10) upon Alpha challenge. Data are presented as mean ± SD. Statistical significance was calculated using one-way ANOVA test (*p<0.05, **p<0.01,***p<0.001, ****p<0.0001).

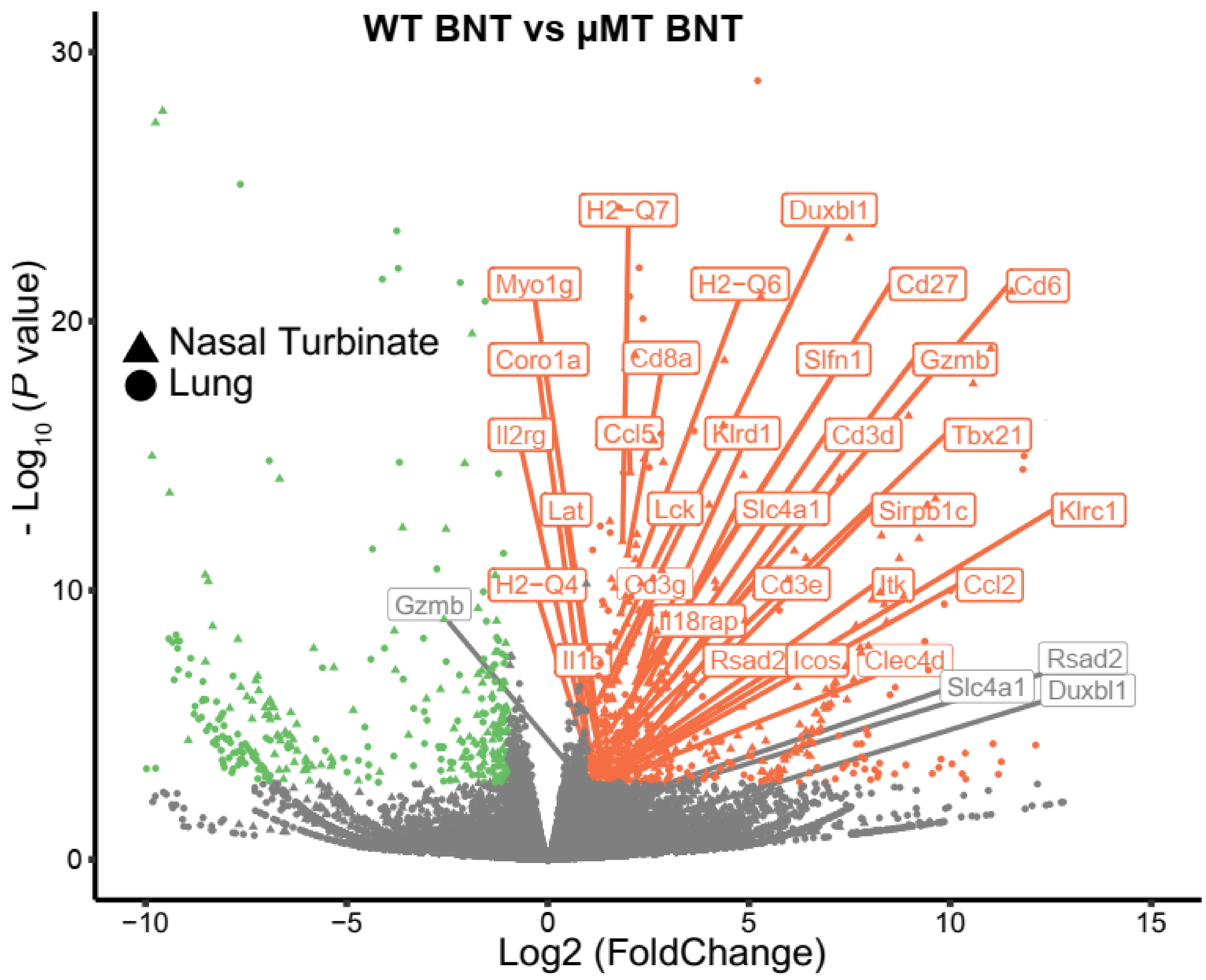

Fig. S4 Volcano plot showing differential expression genes comparing μMT mouse transcriptome to WT in both NT and lung after BNT162b2 vaccination. Genes with orange labels were significantly up regulated in NT or lung of vaccinated μMT mice (adjusted P value < 0.05 and gene expression fold change > 2), scatters with grey labels indicated gene up regulated in both NT and lung of vaccinated WT mice (P value < 0.05 and gene expression fold change > 2).

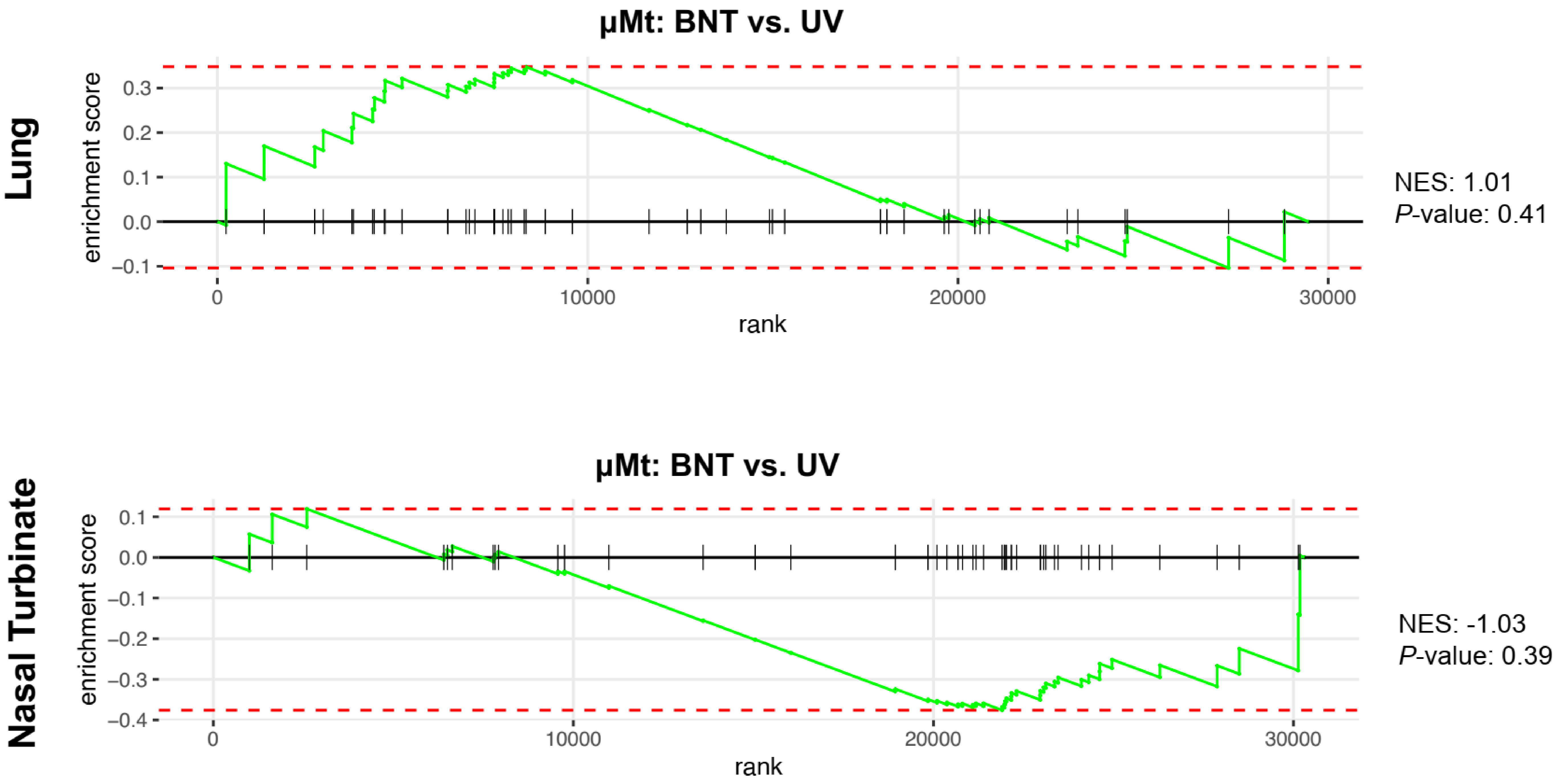

Fig. S5 Gene set enrichment analysis (GSEA) of genes related to complement pattern recognition, complement proteases, complement component, complement receptor and complement regulator in µMT BNT vs. unvaccinated (UV) mice.

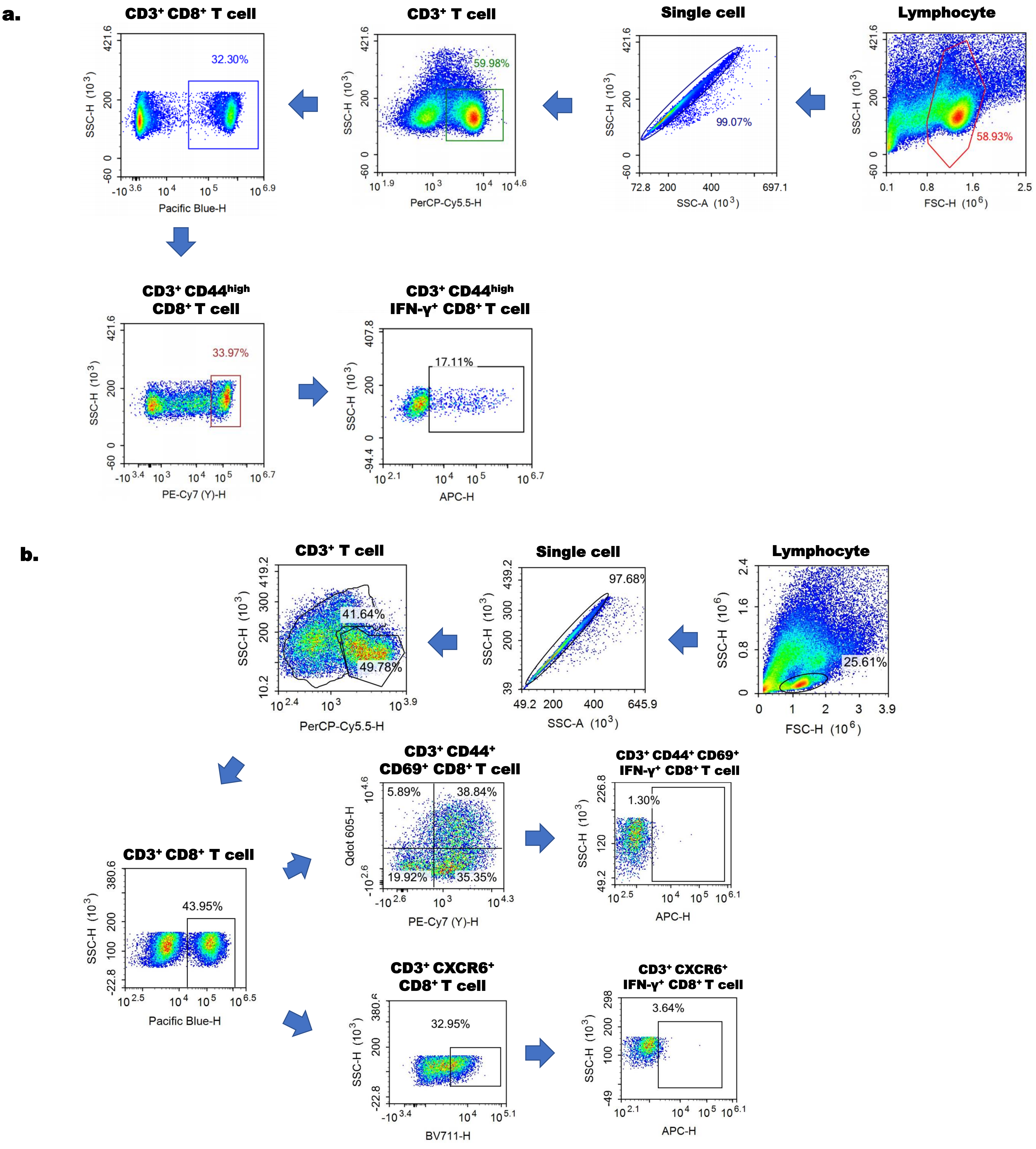

Fig. S6 Gating strategy for detection of (a) CD3⁺ CD8⁺ CD44high IFN-γ⁺ T cells in spleen of vaccinated or unvaccinated C57BL/6J WT and μMT mice after spike protein stimulation; (b) percentage of CD3⁺ CD44⁺ CD69⁺ CD8⁺ ; CD3⁺ CXCR6⁺ CD8⁺ T cells and the corresponding IFN-γ producing T cells in lung tissue from vaccinated or unvaccinated C57BL/6J WT and μMT mice after Alpha infection.

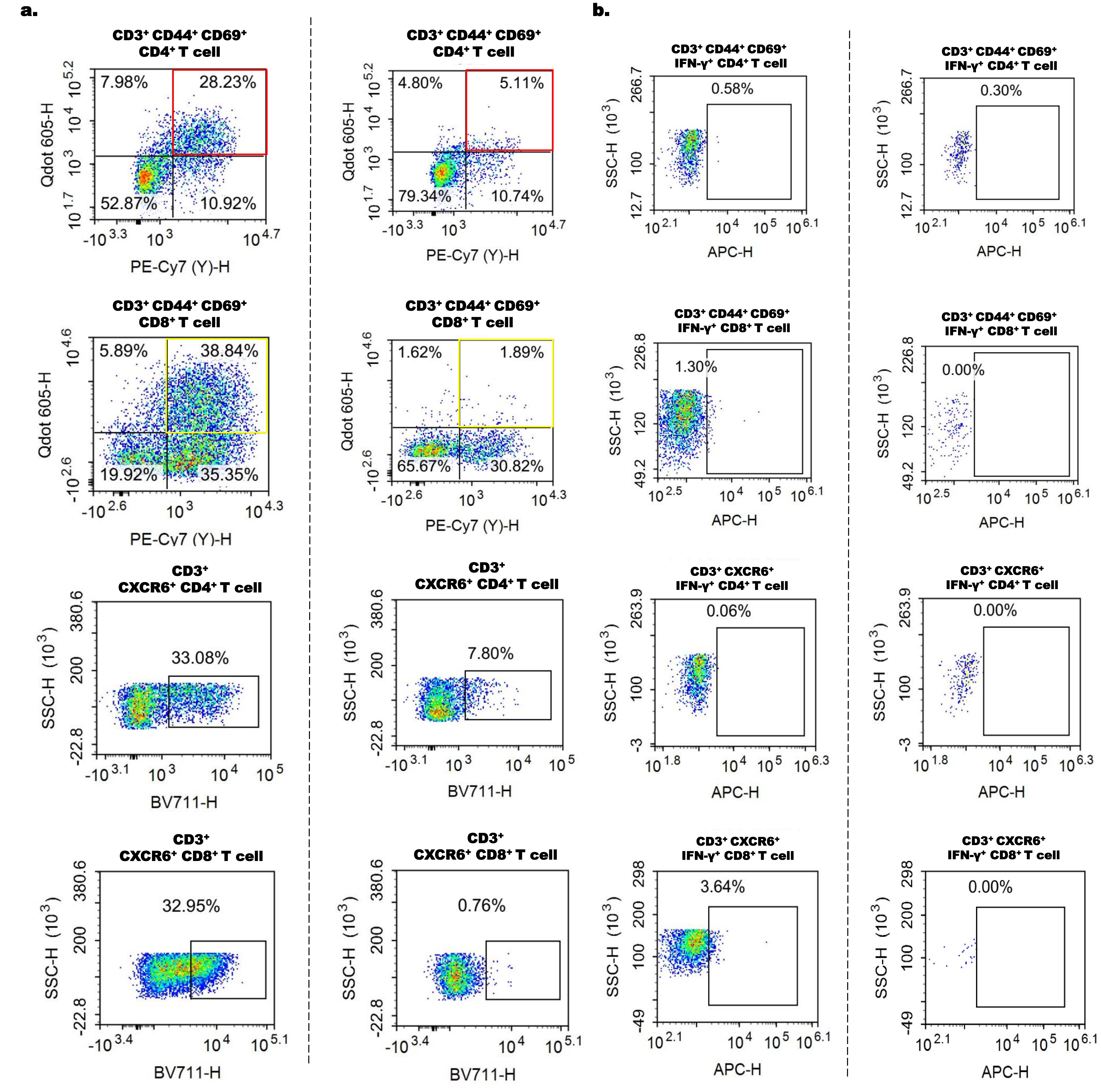

Fig. S7 Representative dot plots showing the percentage of (a) $CD3^{+}CD44^{+}CD69^{+}CD4^{+}$ (Red box); $CD3^{+}CD44^{+}CD69^{+}CD8^{+}$ (Yellow box); $CD3^{+}CXCR6^{+}CD4^{+}$ and $CD3^{+}CXCR6^{+}CD8^{+}$ T cells; (b) $CD3^{+}CD44^{+}CD69^{+}$IFN-$\gamma^{+}CD4^{+}$; $CD3^{+}CD44^{+}CD69^{+}$IFN-$\gamma^{+}CD4^{+}$; $CD3^{+}CXCR6^{+}$IFN-$\gamma^{+}CD4^{+}$ and $CD3^{+}CXCR6^{+}$IFN-$\gamma^{+}CD8^{+}$ T cells in lung tissues from μMT BNT (Left) and μMT unvacc. (Right) mice after 2 d.p.i. of Alpha infection.
