# Supplementary material for: Vaccine-induced protection against SARS-CoV-2 requires IFN-γ-driven cellular immune response
Source: Nat Commun. 2023 Jun 10;14:3440. doi: 10.1038/s41467-023-39096-y (PMC10257169; doi:10.1038/s41467-023-39096-y)
Supplement: Supplementary file 2 — Reporting Summary [file 41467_2023_39096_MOESM2_ESM.pdf]

nature portfolio

Corresponding author(s): Jian-dong Huang

Last updated by author(s): Apr 15, 2023

# Reporting Summary

Nature Portfolio wishes to improve the reproducibility of the work that we publish. This form provides structure for consistency and transparency in reporting. For further information on Nature Portfolio policies, see our Editorial Policies and the Editorial Policy Checklist.

## Statistics

For all statistical analyses, confirm that the following items are present in the figure legend, table legend, main text, or Methods section.

| n/a | Confirmed | |
|---|---|---|
| ☐ | ☒ | The exact sample size (*n*) for each experimental group/condition, given as a discrete number and unit of measurement |
| ☐ | ☒ | A statement on whether measurements were taken from distinct samples or whether the same sample was measured repeatedly |
| ☐ | ☒ | The statistical test(s) used AND whether they are one- or two-sided<br>*Only common tests should be described solely by name; describe more complex techniques in the Methods section.* |
| ☒ | ☐ | A description of all covariates tested |
| ☐ | ☒ | A description of any assumptions or corrections, such as tests of normality and adjustment for multiple comparisons |
| ☐ | ☒ | A full description of the statistical parameters including central tendency (e.g. means) or other basic estimates (e.g. regression coefficient) AND variation (e.g. standard deviation) or associated estimates of uncertainty (e.g. confidence intervals) |
| ☐ | ☒ | For null hypothesis testing, the test statistic (e.g. *F*, *t*, *r*) with confidence intervals, effect sizes, degrees of freedom and *P* value noted<br>*Give P values as exact values whenever suitable.* |
| ☒ | ☐ | For Bayesian analysis, information on the choice of priors and Markov chain Monte Carlo settings |
| ☒ | ☐ | For hierarchical and complex designs, identification of the appropriate level for tests and full reporting of outcomes |
| ☒ | ☐ | Estimates of effect sizes (e.g. Cohen's *d*, Pearson's *r*), indicating how they were calculated |

*Our web collection on statistics for biologists contains articles on many of the points above.*

## Software and code

Policy information about availability of computer code

| | |
|---|---|
| Data collection | H&E stain images were acquired using Vectra Polaris automated quantitative pathology imaging system 460 (Perkin Elmer).<br>LightCycler96 (version SW1.1) was used to collect qPCR data.<br>Flow cytometry data were acquired on a ACEA NovoCyte Quanteon flow cytometer (Agilent). |
| Data analysis | For RNA-seq analysis, Salmon was used to quantify mouse and SARS-CoV-2 gene expression. SARS-CoV-2 gene expression profile was calculated from un-mapped reads which were retrieved 477 after executing alignment to mouse reference genome by STAR. Gene differential expressions analysis were carried out by DESeq2. ClusterProfiler was used for immune and viral process related gene ontologies and KEGG pathway enrichment analysis. Cell type functional enrichment were conducted by xCell using moue gene expression profile. T cell and B cell receptor repertoire analysis from clean data was conducted by MiXCR. NovoExpress® Software was used to analyze the flow cytometry data. For other data analysis, GraphPad Prism v.8.0 was used. |

For manuscripts utilizing custom algorithms or software that are central to the research but not yet described in published literature, software must be made available to editors and reviewers. We strongly encourage code deposition in a community repository (e.g. GitHub). See the Nature Portfolio guidelines for submitting code & software for further information.

## Data

Policy information about availability of data

All manuscripts must include a data availability statement. This statement should provide the following information, where applicable:

- Accession codes, unique identifiers, or web links for publicly available datasets
- A description of any restrictions on data availability
- For clinical datasets or third party data, please ensure that the statement adheres to our policy

Source data are provided with this paper in Source Data file. Raw bulk RNA-seq data have been deposited in the NCBI Sequence Read Archive .

## Human research participants

Policy information about studies involving human research participants and Sex and Gender in Research.

| | |
|---|---|
| Reporting on sex and gender | N/A |
| Population characteristics | N/A |
| Recruitment | N/A |
| Ethics oversight | N/A |

Note that full information on the approval of the study protocol must also be provided in the manuscript.

# Field-specific reporting

Please select the one below that is the best fit for your research. If you are not sure, read the appropriate sections before making your selection.

☒ Life sciences ☐ Behavioural & social sciences ☐ Ecological, evolutionary & environmental sciences

For a reference copy of the document with all sections, see nature.com/documents/nr-reporting-summary-flat.pdf

# Life sciences study design

All studies must disclose on these points even when the disclosure is negative.

| | |
|---|---|
| Sample size | Sample size is chosen based on the standard of the corresponding field. This typically resulted in a minimum sample size of n=5-8. For some experiments with high variability, sample size was increased. |
| Data exclusions | No data were excluded. |
| Replication | All experiments were repeated at least twice on two different days. Similar findings were obtained from all repeats. |
| Randomization | The animal used in animal experiments were randomly allocated into different groups. |
| Blinding | Mice were randomized into different groups before any procedure and the university animal care committee requires detailed cage labelling. The investigators were not blinded to group allocation. |

# Reporting for specific materials, systems and methods

We require information from authors about some types of materials, experimental systems and methods used in many studies. Here, indicate whether each material, system or method listed is relevant to your study. If you are not sure if a list item applies to your research, read the appropriate section before selecting a response.

## Materials & experimental systems

| n/a | Involved in the study | |
|---|---|---|
| ☐ | ☒ | Antibodies |
| ☐ | ☒ | Eukaryotic cell lines |
| ☒ | ☐ | Palaeontology and archaeology |
| ☐ | ☒ | Animals and other organisms |
| ☒ | ☐ | Clinical data |
| ☒ | ☐ | Dual use research of concern |

## Methods

| n/a | Involved in the study | |
|---|---|---|
| ☒ | ☐ | ChIP-seq |
| ☐ | ☒ | Flow cytometry |
| ☒ | ☐ | MRI-based neuroimaging |

# Antibodies

| | |
|---|---|
| Antibodies used | Goat anti-Mouse IgG (H+L), HRP (62-6520, Thermo Fisher Scientific);<br>anti-CD3 PerCP-Cy5.5 (BioLegend 100218);<br>anti-CD4 FITC (BioLegend 100406);<br>anti-CD8 BV421 (BioLegend 100738);<br>anti-CD44 423 PE-Cy7 (BioLegend 103030);<br>anti–IFN-γ-APC (BioLegend 505810);<br>anti–IL-4-PE (BioLegend 372208);<br>anti-CD127 APC (BioLegend 135012);<br>anti-KLRG1 PE (BioLegend 138408);<br>anti-mouse CD4 (BioXcell InVivoMab Clone GK 1.5);<br>anti-mouse CD8 (BioXcell InVivoMab Clone 2.43);<br>anti-mouse IFN-γ (BioXcell InVivoMab Clone XMG1.2);<br>anti-CD69 BV605 (BioLegend 104530);<br>anti-CXCR6 BV711 (BioLegend 151111);<br>anti–Granzyme B APC (BioLegend 372208). |
| Validation | Commercial primary antibodies were validated by the manufacturers and validation statements are available on the manufacturer's website.<br>Statement from BioLegend:<br>Specificity testing of 1-3 target cell types with either single- or multi-color analysis (including positive and negative cell types).<br>Once specificity is confirmed, each new lot must perform with similar intensity to the in-date reference lot. Brightness (MFI) is evaluated from both positive and negative populations. Each lot product is validated by QC testing with a series of titration dilutions.<br>Statement from BioXCell:<br>Our InVivoMAb™ antibodies are specifically formulated for in vivo use. They feature greater than 95% purity, ultra-low endotoxin levels, and are preservative, stabilizer, and carrier protein-free. Many of our InVivoMAb™ antibodies can also be used for in vitro applications including Western blotting, ELISA, flow cytometric analysis, immunofluorescence, immunohistochemistry, and immunoprecipitation. All InVivoMAb™ products are screened for purity and integrity via SDS-PAGE and guaranteed to contain less than 2 endotoxin units per milligram. |

# Eukaryotic cell lines

Policy information about cell lines and Sex and Gender in Research

| | |
|---|---|
| Cell line source(s) | VeroE6 cells were obtained from ATCC. |
| Authentication | The cell line was not authenticated. |
| Mycoplasma contamination | The cell line has been recently tested negative for mycoplasma contamination. |
| Commonly misidentified lines<br>(See ICLAC register) | No commonly misidentified cell lines were used in this study. |

# Animals and other research organisms

Policy information about studies involving animals; ARRIVE guidelines recommended for reporting animal research, and Sex and Gender in Research

| | |
|---|---|
| Laboratory animals | Six to 12-week-old C57Bl/6J (WT), B6.129S2-Ighmtm1Cgn/J (µMT), and B6.Cg-Tg(K18-ACE2)2Prlmn/J (K18-hACE2) were purchased from the Jackson Laboratory and were subsequently bred and housed at the Center for Comparative Medicine Research (CCMR) of University of Hong Kong. K18-hACE2 µMT mice were obtained by cross-breeding of B6.Cg-Tg(K18-ACE2)2Prlmn/J mice with B6.129S2-387 Ighmtm1Cgn/J (µMT) mice. |
| Wild animals | No wild animals were involved in this study. |
| Reporting on sex | Gender-matched mice were randomized into different experimental groups. |

| | |
|---|---|
| Field-collected samples | No field-collected samples are involved in this study. |
| Ethics oversight | The use of animals has complied with all relevant ethical regulations and was approved by the Committee on the Use of Live Animals in Teaching and Research of The University of Hong Kong |

Note that full information on the approval of the study protocol must also be provided in the manuscript.

# Flow Cytometry

## Plots

Confirm that:

- [x] The axis labels state the marker and fluorochrome used (e.g. CD4-FITC).
- [x] The axis scales are clearly visible. Include numbers along axes only for bottom left plot of group (a 'group' is an analysis of identical markers).
- [x] All plots are contour plots with outliers or pseudocolor plots.
- [x] A numerical value for number of cells or percentage (with statistics) is provided.

## Methodology

| | |
|---|---|
| Sample preparation | Splenocytes were isolated 21 days after the second immunization, plated in 24-well plates containing spike protein trimer (50 µg/mL) at 37 °C for 16–18 h in the presence of Brefeldin A (5 µg/mL) for the last 4 h. The cells were then stained with fluorophore-conjugated surface marker-specific antibodies, fixed, permeabilized, washed in Perm/Wash buffer (BD Biosciences), and then stained with anti–IFN-γ-PE (BioLegend), anti–IL-4-APC (BioLegend),in Perm/Wash buffer (BD Biosciences) for 20 min at room temperature, washed twice in Perm/Wash buffer, and suspended in PBS.<br>For lung tissues harvested after viral infection, the single cell suspension was obtained by digesting the samples with Collagenase/Hyaluronidase (StemCell) and DNase I (StemCell) solution. The samples were then stained with anti-CD3 PerCP-Cy5.5 (BioLegend), anti-CD4 FITC (BioLegend), anti-CD8 BV421 (BioLegend), anti-CD44 PE-Cy7 (BioLegend), anti-CD69 BV605 (BioLegend) and anti-CXCR6 BV711 (BioLegend), followed by fixation of 12 h. The cells were subsequently permeabilized with Cytofix/Cytoperm (BD Biosciences), washed in Perm/Wash buffer (BD Biosciences), and then stained with anti–IFN-γ APC (BioLegend) and anti–Granzyme B APC (BioLegend) in Perm/Wash buffer (BD Biosciences) for 20 min at room temperature, washed twice in Perm/Wash buffer, and suspended in PBS. |
| Instrument | Data were acquired on a ACEA NovoCyte Quanteon flow cytometer (Agilent). |
| Software | The data were analyzed using NovoExpress® Software. |
| Cell population abundance | Cell population abundance shown in each figure. |
| Gating strategy | The gating strategy was demonstrated in Supplementary Figure 5.<br>The cells were gated as:<br>Splenic CD8+ memory cells: CD3+CD8+CD44highCD127+KLRG1-<br>Splenic CD8+ terminal effector cells: CD3+CD8+CD44highCD127-KLRG1+<br>Lung CD69+ TRM cells: CD3+CD4+CD44+CD69+, CD3+CD8+CD44+CD69+<br>Lung CXCR6+ TRM cells: CD3+CD4+CD44+CXCR6+, CD3+CD8+CD44+CXCR6+ |

- [x] Tick this box to confirm that a figure exemplifying the gating strategy is provided in the Supplementary Information.
